# Force-Induced Spiropyran–Merocyanine Interconversion: An Enforced Geometry Optimization Study

**DOI:** 10.3390/ijms27219767

**Published:** 2026-10-30

**Authors:** Agnieszka Brzyska

**Affiliations:** Jerzy Haber Institute of Catalysis and Surface Chemistry, Polish Academy of Sciences, Niezapominajek 8, 30-239 Cracow, Poland; agnieszka.brzyska@ikifp.edu.pl

**Keywords:** spiropyran, merocyanine, mechanochemistry, enforced geometry optimization, substituent effects, solvent effects

## Abstract

Mechanically induced spiropyran (SP)–merocyanine (MC) interconversion was examined using the enforced geometry optimization (EGO) method at the B3LYP/6-311G(d,p) level. The calculations followed a complete mechanical cycle for the SP–CH_3_ model: tensile opening of the spiro ring, force removal, and subsequent compression. This allowed both SP → MC conversion and recovery of the closed SP structure to be considered within the same computational framework. Across eleven spiropyran derivatives, the first observed opening forces show a clear overall Hammett-like trend, with strongly electron-withdrawing substituents opening earlier and strongly electron-donating substituents opening later. A closer analysis of the H-, NH_2_-, and Cl-substituted systems showed that C–O cleavage is accompanied by loss of the localized spiro bond, Wiberg bond indices falling to values near zero, charge redistribution, and narrowing of the HOMO–LUMO gap. Calculations in the gas phase and in thirteen COSMO environments revealed a further feature: a low-force geometric reorganization precedes bond cleavage. The solvent also influences which relaxed MC conformer is reached, giving TTC, CTC, CCC, or CCT structures. Neither the calculated ring-opening point nor the final MC conformation is determined solely by the dielectric constant.

## 1. Introduction

Mechanical force can do more than deform a molecule. If transmitted along a suitable molecular coordinate, it can alter the geometry enough to initiate a chemical transformation. In polymeric materials, such forces may be carried through covalent chains to mechanophores—molecular units that respond to loading by changing their structure, reactivity, or optical properties [1,2,3,4]. Spiropyran is one of the most extensively studied examples. Force-induced opening of its ring produces the colored merocyanine form, which has made spiropyran a useful probe of local stress and deformation in polymeric materials [1,5].

The closed spiropyran (SP) consists of two heterocyclic fragments joined at a tetrahedral spiro carbon. This geometry interrupts conjugation between the two molecular halves. Cleavage of the C_spiro_–O bond produces merocyanine (MC), with a more extended molecular framework and a conjugated electronic system [5]. The SP–MC transformation can be induced by light, temperature, changes in the chemical environment, or mechanical force.

The mechanical response of spiropyran is not determined by the intrinsic strength of the C_spiro_–O bond alone. The chemical structure defines the underlying energy landscape, but the way in which force is introduced into the molecule determines how effectively that landscape is perturbed along the ring-opening coordinate. Gossweiler et al. [6] showed that changing the connectivity of the force-bearing groups alters the force dependence of spiropyran ring-opening kinetics. Closely related structures may therefore respond differently to the same nominal load if the force is transmitted through a different part of the molecular framework.

Substitution changes the response in another way. Barbee et al. [7] examined H-, Br-, and NO_2_-substituted spiropyrans by single-molecule force spectroscopy and found that the more electron-withdrawing substituent required less force to reach a specified ring-opening rate. Their force-dependent kinetic data were consistent with a polar, heterolytic, dissociative ring-opening process. The electronic effect of a substituent, however, cannot be considered independently of force transmission. Kempe et al. [8] showed that both the substitution pattern and the position of the force-bearing attachment influence the energetics of mechanically induced ring opening. Multiscale modeling likewise indicates that the surrounding polymer architecture can modify how efficiently load is transferred to the mechanophore [9].

Force acts on a chemically defined reaction landscape through a specific mechanical pathway, and the observed kinetics reflect both. Tensile stress accelerates SP → MC conversion while slowing the reverse MC → SP process [10]. Internal compressive stress, in contrast, can favor the contraction associated with MC → SP ring closure and oppose the expansion required for SP → MC opening [11]. Ring-opening kinetics are consequently shaped by the electronic structure of the mechanophore and by the direction and efficiency with which force is transmitted through it.

Thermal and photochemical SP–MC conversion has been studied in considerable detail. Quantum-chemical studies have described SP–MC conversion pathways and substituent- and solvent-dependent energetics [12,13,14]. Ultrafast spectroscopic studies have followed the formation and subsequent isomerization of open merocyanine species [15,16,17]. These studies provide a detailed picture of the electronic and conformational landscape of the system. A mechanically selected pathway, however, need not follow the same sequence of structures.

Single-molecule force spectroscopy gives direct access to force-dependent reaction kinetics, but it does not reveal the complete structural sequence preceding bond cleavage. A measured force–rate relationship cannot show whether the closed SP framework undergoes a distinct reorganization before the C_spiro_–O bond breaks, nor does it identify the geometry reached immediately after opening. Separating electronic and mechanical effects is also difficult when substitution, attachment position, and molecular environment vary at the same time [6,7,8,9,10,11]. In a polymer, the local force acting on the mechanophore is additionally determined by how stress is transferred through the surrounding chain or network [9].

The molecular environment adds another variable. Solvent polarity has been shown to alter the force-dependent kinetics of spiropyran opening [18], while earlier calculations demonstrated substantial solvent effects on SP–MC energetics and electronic structure [12,14]. Less is known about how the environment modifies the mechanically selected structural pathway itself, particularly before bond cleavage and during relaxation of the open MC product.

Enforced geometry optimization (EGO) is well suited to examining these questions because the atoms through which force is applied, as well as its magnitude and direction, are defined explicitly [19,20]. External forces act on selected atomic nuclei while the remaining molecular coordinates are optimized. Repeating the calculation at successive force values provides a sequence of optimized structures along a force-modified pathway. EGO has previously been used to study force-induced structural transformations and mechanically generated metastable states [19,20], and it has also been applied in force-spectroscopy simulations and in studies of mechanically induced conformational changes in carbohydrate systems [21,22,23,24]. Related computational approaches have addressed mechanochemical ring opening, covalent-bond strength, and reaction-path searches [25,26,27]. EGO does not reproduce single-molecule force spectroscopy directly. Experimental transition forces are kinetic observables and depend on loading conditions and timescale [6,18], whereas EGO follows optimized molecular structures under prescribed static forces [19,20]. Force spectroscopy measures how the reaction rate responds to load; EGO can resolve how molecular geometry and electronic structure change as a defined load is transmitted through a specified molecular pathway.

**Figure 1:**
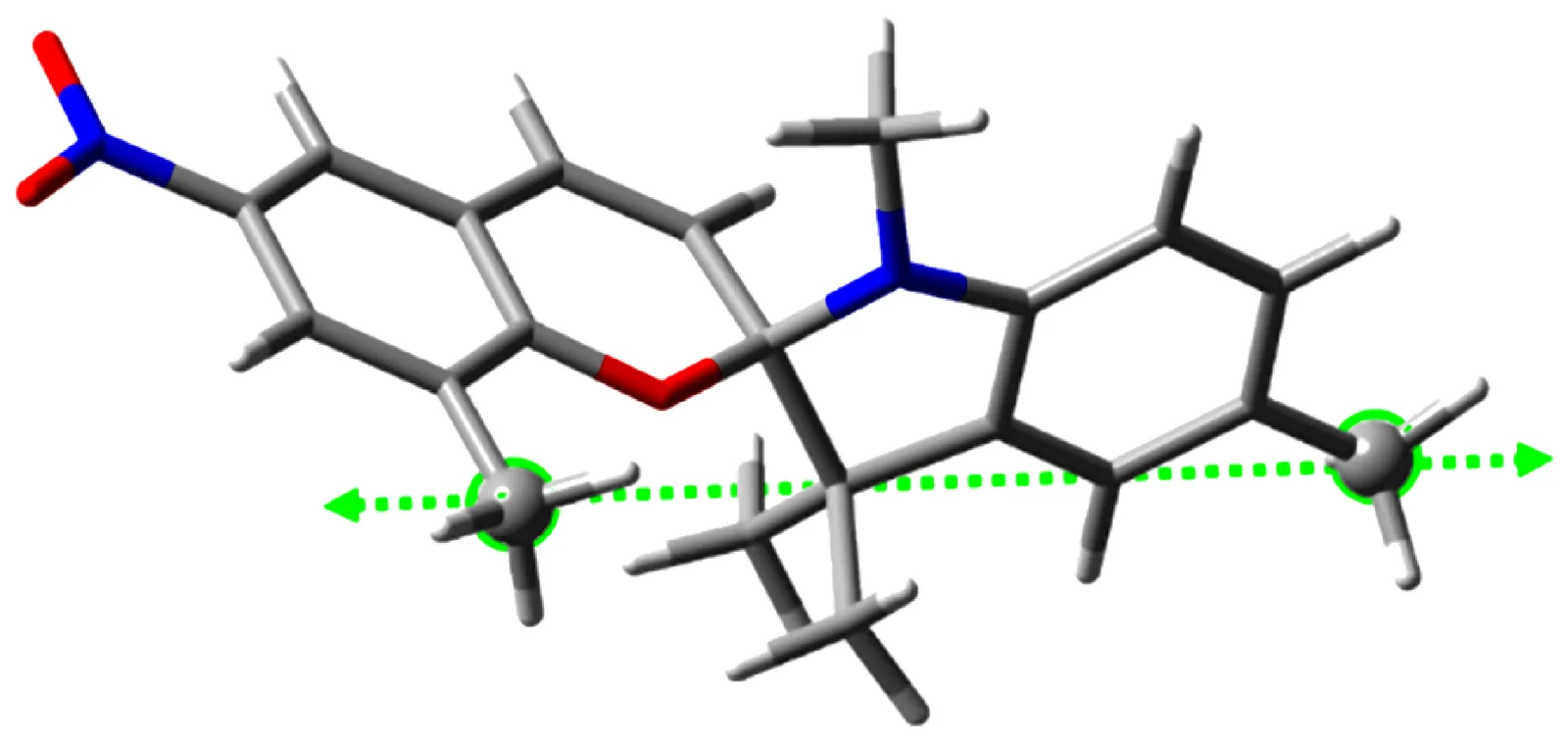
Spiropyran molecule—application of external tensile forces in EGO calculations. Green dotted arrows indicate the directions of the tensile forces applied to the atoms highlighted with green circles.

The mechanical boundary conditions used in the present calculations are shown in Figure 1. Equal and oppositely directed forces were applied to the selected force-bearing atoms, transmitting tensile load through the spiropyran framework toward the mechanically active C_spiro_–O bond. The choice of these atoms defines how the load enters the molecule. The resulting response depends on both connectivity and the direction of force transmission [6,8].

The calculations address several questions that remain difficult to separate experimentally. Does the closed SP framework reorganize before the spiro C–O bond breaks? If different substituents are examined under the same force-bearing geometry, does their response follow a simple electronic trend? What happens to the mechanically generated MC after the external force is removed, and can reverse loading recover the closed SP minimum? The influence of the molecular environment was examined in the same framework, with particular attention to the structures reached before and after bond cleavage.

SP–MC interconversion in the SP–CH_3_ model was followed during tensile loading, zero-force relaxation, and subsequent compression. Complete stretching scans were obtained for eleven substituted spiropyrans under a common loading protocol. Three members of this series—H, NH_2_, and Cl—were selected for a more detailed gas-phase analysis. Their closed structures and force-opened structures were obtained at 0.065 a.u. (5.36 nN), and products relaxed after removal of the force were characterized using molecular geometry, NBO analysis, Wiberg bond indices, CHELP electrostatic charges, dipole moments, and frontier-orbital energies. The calculations at 0.065 a.u. characterize products that are already open under the same applied load and are therefore distinct from the individual scans used to locate the ring-opening force intervals.

Solvent effects were examined separately in the gas phase and in thirteen COSMO continuum environments ranging from weakly to strongly polar media. For each environment, the closed SP structure was compared with the structure obtained at 0.065 a.u. (5.36 nN) and with the product after zero-force relaxation. Structural and electronic descriptors were analyzed for all three states. The EGO force values reported here identify structural changes along specific optimized pathways under prescribed mechanical loading. They should not be interpreted as experimental kinetic activation forces.

## 2. Results and Discussion

### 2.1. Force-Induced Ring Opening of Spiropyran

**Figure 2:**
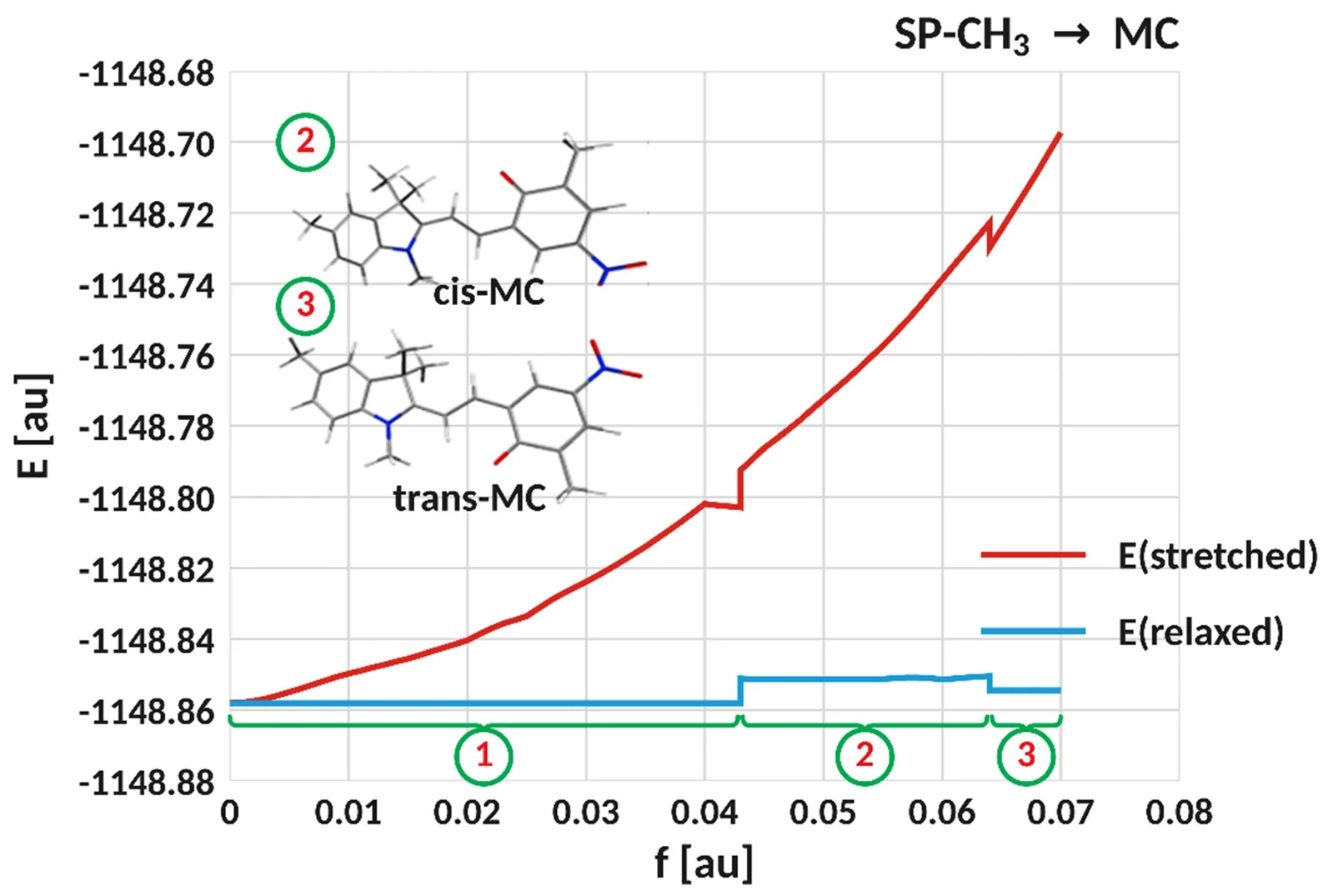
Molecular-energy profiles obtained during EGO stretching of the SP-CH_3_ model and subsequent zero-force relaxation. The profiles include additional calculations between f = 0 and 0.010 a.u. E(relaxed) denotes energies after removal of the external force; the horizontal coordinate identifies the preceding tensile load. Force increment: 0.001 a.u. (0.082 nN). Plotted electronic energies exclude the external-force work term and are not activation barriers.

**Scheme 1:**
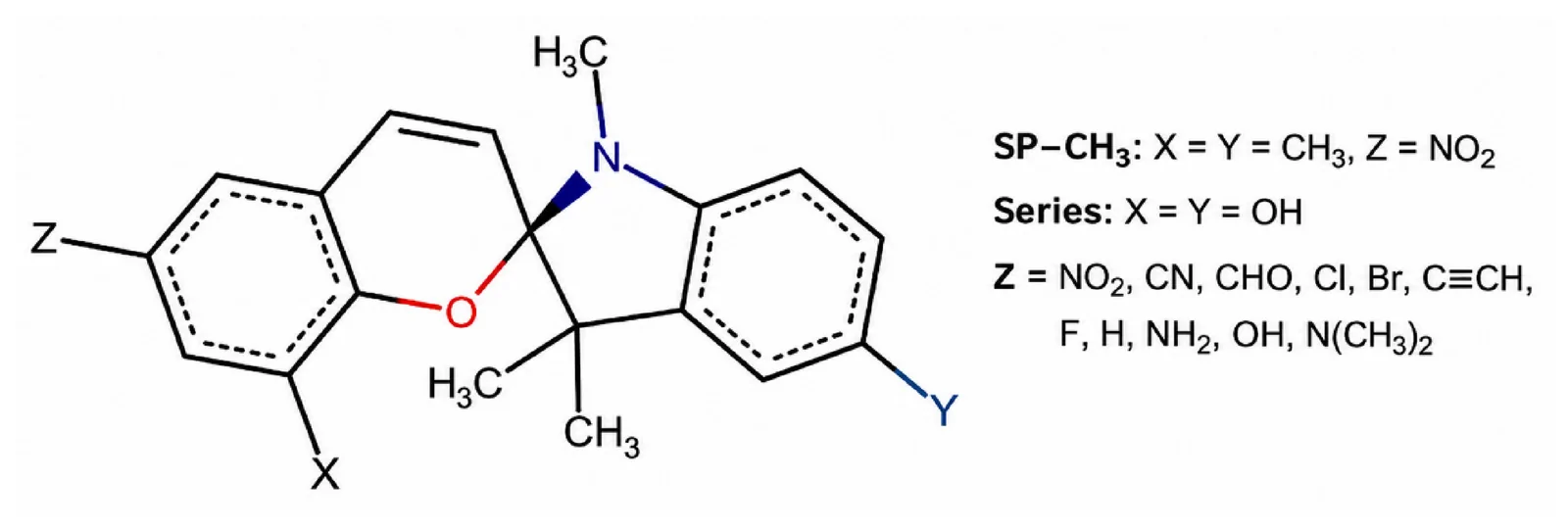
Molecular structures and substitution patterns of the investigated spiropyran models. The SP–CH_3_ model (X = Y = CH_3_, Z = NO_2_) is used in Figure 2, Figure 3 and Figure 4. For the eleven-member substituent series (Figure 6 and Table S7), X = Y = OH and Z is varied; SP–OH in Figure 5 and Figure 7 denotes its Z = NO_2_ member.

Figure 2 shows how the SP–CH_3_ model (X = Y = CH_3_, Z = NO_2_; Scheme 1) responds to tensile loading and subsequent relaxation at zero force. At low force, the molecule deforms progressively but remains in the closed SP form. Further stretching eventually cleaves the C_spiro_–O bond and generates an open merocyanine structure. For this system, the first geometry-confirmed SP → MC conversion occurs in the approximate interval 0.040–0.045 a.u. (3.30–3.71 nN). In the corrected energy profiles, an upward step near 0.043 a.u. accompanies entry into the cisoid-MC-like regime. A further downward step near 0.064 a.u. separates this regime from the transoid-MC-like structures. The relaxed MC branch remains above the initial closed-SP energy, consistent with the SP–CH_3_ endpoint comparison in Figure 4b. Beyond this point, continued loading mainly changes the geometry of a product that is already open. Initial C_spiro_–O cleavage should therefore be separated conceptually from the later conformational reorganization of MC. This distinction is consistent with the known conformational complexity of merocyanine. Quantum-chemical studies of SP↔MC isomerization describe a multistep landscape containing several open conformers separated mainly by rotations within the methine bridge [13]. Ion-mobility measurements provide experimental support for distinct cisoid and transoid MC structures [28], while ultrafast studies show sequential interconversion among open merocyanine species [16,17]. In toluene, TTC and TTT isomers are prominent, and additional CTC/CTT-type species can be reached photochemically [29]. The structures obtained immediately after C_spiro_–O cleavage in the present EGO scan are therefore best described, qualitatively, as cis- or cisoid-MC-like. The more extended structures reached under further tension are transoid-MC-like. Mechanical loading thus does more than break the bond: once the ring has opened, continued tension biases the molecule toward geometries with a larger end-to-end extension. The mechanically selected MC pathway need not be identical to the thermal or photochemical one. At still higher tensile forces, the response is no longer productive SP → MC switching. Structural distortion and bond damage appear in the approximately 0.07–0.08 a.u. (5.77–6.59 nN) range. Three regimes can therefore be distinguished in Figure 2. At low force, closed SP is deformed; at intermediate force, SP → MC conversion is followed by conformational reorganization of MC; and at the highest forces, structural degradation dominates. The upper limit is specific to the molecular structure, force-bearing geometry, and computational protocol used here and should not be regarded as a universal rupture force for spiropyran.

High-force structural degradation was identified from bond rupture and severe structural distortion outside the intended spiro C–O switching pathway, accompanied by loss of SCF convergence. The nature of the structural changes associated with degradation is system-dependent: both the bonds affected and the extent of structural damage vary among the molecular systems. A representative example for SP–CH_3_, with the corresponding structural comparison, is provided in the Supporting Information (Section S8, Figure S4).

**Figure 3:**
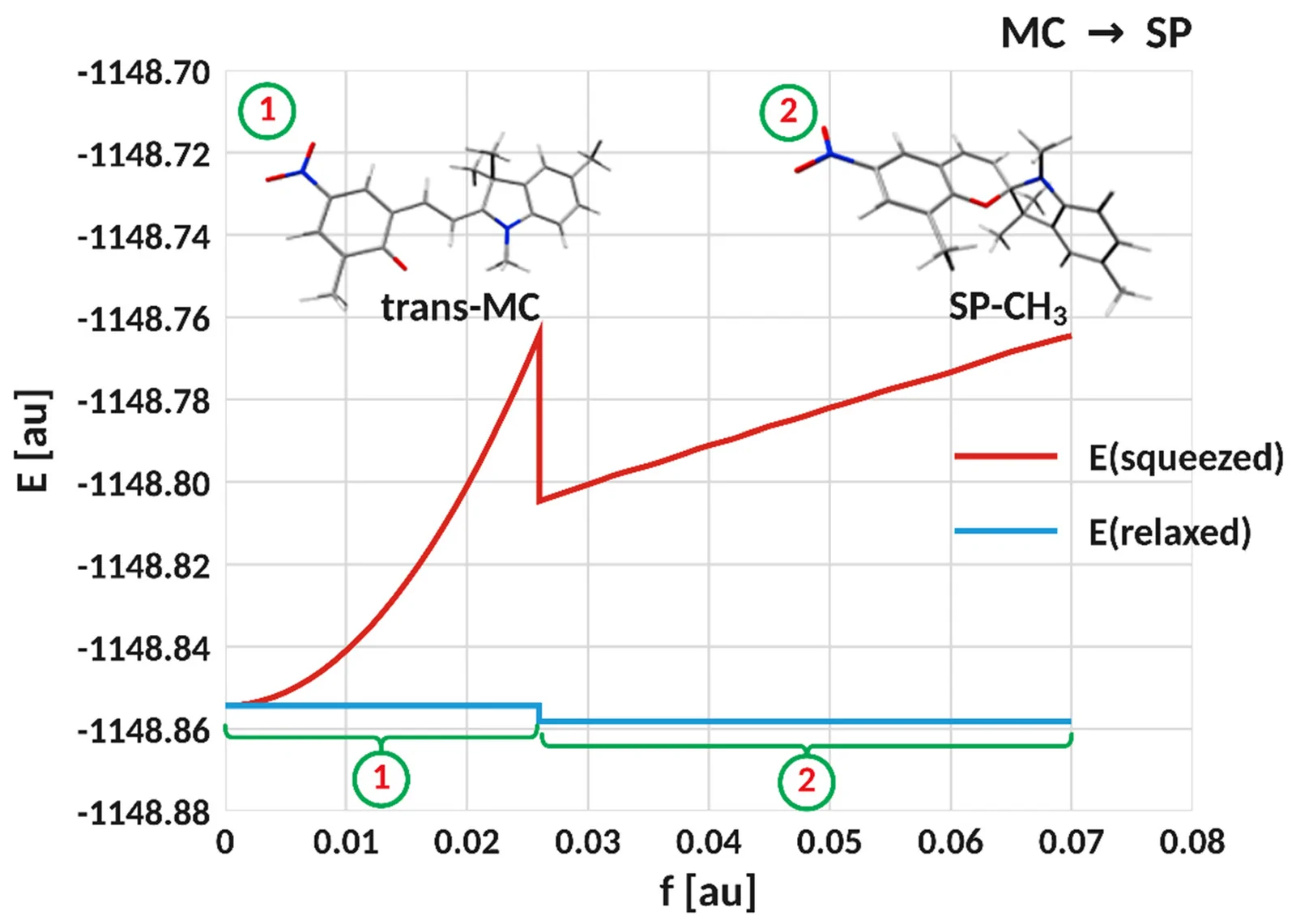
Molecular-energy profiles obtained during EGO compression and relaxation of MC. Compression started from the relaxed MC structure generated during stretching. Force increment: 0.001 a.u. (0.082 nN). E(relaxed) denotes zero-force energies; the horizontal coordinate identifies the preceding compressive load. The loaded and relaxed profiles share the same zero-force MC reference energy. Plotted electronic energies exclude the external-force work term and are not activation barriers.

After tensile loading, the generated MC structure was reoptimized with the external force completely removed. The zero-force optimization converged to an open MC structure. This zero-force-relaxed MC geometry then served as the starting point for compression (Figure 3). Increasing the compressive force restored the closed SP framework near 0.026 a.u. (2.14 nN) in the corrected scan (Figure 3). The drop in the loaded-state energy is accompanied by a shift of the zero-force-relaxed energy from the MC branch to the lower-energy SP branch. The force magnitude associated with MC → SP closure is therefore substantially lower than that for tensile SP → MC opening, approximately 0.040–0.045 a.u. (3.30–3.71 nN). This difference in force magnitude does not directly quantify the relative MC → SP and SP → MC activation barriers. The two EGO trajectories start from different local minima, and tension and compression couple differently to molecular extension. Tension favors access to the more extended MC state; compression, by contrast, is geometrically aligned with contraction toward compact SP. The corresponding transition forces therefore need not be symmetric even though both processes involve the same SP/MC pair. Experimental observations strongly support this directional character of mechanochemical coupling. In spiropyran-containing polyurethane, Kim et al. showed that tensile stress increases the SP → MC rate while decreasing the MC → SP rate; at 50 MPa, the forward rate increased by 110%, whereas the reverse rate decreased by 65% [10]. The compression calculation is even more directly related to the work of Janissen and Filonenko. During PMMA vitrification, they found that internal compressive stress lowered the measured activation energy for contraction-associated MC → SP ring closure from 147 to 79 kJ mol^−1^, a reduction of 68 kJ mol^−1^ [11]. In the same study, compressive stress raised the barrier for the expansion-associated SP → MC process. Their conclusion—that compression promotes molecular contraction while opposing expansion—is qualitatively consistent with the lower compression force found here. The comparison nevertheless has clear limits. Direct longitudinal compression of an isolated mechanophore is not a standard experimental analog of polymer stretching; under macroscopic compression, real polymer chains may buckle, reorganize, or transfer stress through their surroundings instead of exerting a perfectly collinear pushing force. The EGO compression calculation is therefore best regarded as a controlled probe of the force-modified molecular energy landscape. The internal-stress experiments of Janissen and Filonenko support the direction of the effect, rather than providing a one-to-one realization of the EGO loading geometry.

The high-force region adds another useful observation. During compression, structural degradation is again observed near approximately 0.07 a.u. (5.77 nN), broadly similar to the upper destructive range found for stretching. The desired structural transition, however, is strongly direction-dependent. This suggests that the lower boundary of useful mechanochemical response is governed mainly by coupling of the applied force to the SP ⇄ MC coordinate, whereas severe degradation at high force reflects more general overstressing of the molecular framework. The calculated sequence is therefore SP → MC (tension) → MC (f = 0 relaxation) → SP (compression).

**Figure 4:**
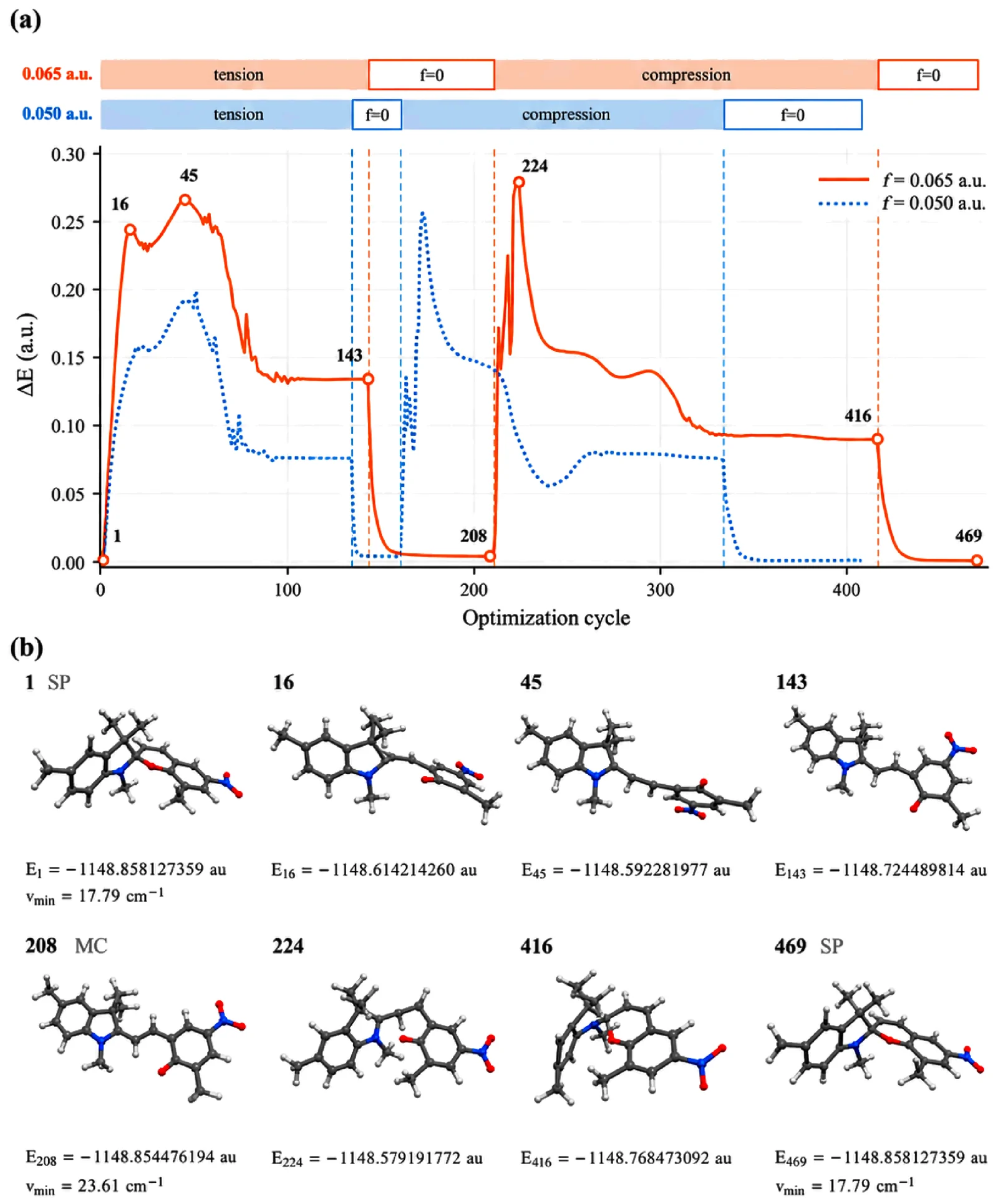
EGO stretching–relaxation–compression–relaxation cycle of the SP–CH_3_ model, computed at B3LYP/6-311G(d,p). (**a**) Algorithmic reaction paths for constant applied forces of 0.065 a.u. (≈5.4 nN; solid line) and 0.050 a.u. (≈4.1 nN; dotted line). The same force magnitude was used for tension and compression. The bars above the plot show the sequence of loading stages for each path: tension, relaxation at zero force (f = 0), compression, and final relaxation at f = 0. Dashed vertical lines mark the stage boundaries. (**b**) Molecular structures along the 0.065 a.u. path, labeled by optimization cycle, with their total electronic energies E. Plotted electronic energies exclude the external-force work term and are not activation barriers.

Figure 4 extends the force scans in Figure 2 and Figure 3 by following complete loading–relaxation cycles at two fixed force magnitudes, f = 0.050 and 0.065 a.u. (4.12 and 5.36 nN). The selected force is maintained during both the tensile and compressive stages. During each relaxation stage, it is removed completely, so that f = 0. Both values lie above the tensile opening interval established independently in Figure 2 and below the observed high-force structural degradation region.

The trajectory at f = 0.050 a.u. (4.12 nN) samples the lower part of the non-destructive activated regime, relatively close to the SP → MC opening interval. The f = 0.065 a.u. (5.36 nN) trajectory applies a substantially larger load and approaches the degradation region. Yet the qualitative sequence is the same in both cases: tension generates MC, force removal leaves an open relaxed product, reverse loading restores SP, and the final zero-force relaxation returns the system to the closed state. Calculated reversibility is therefore not tied to one arbitrarily chosen force value. The full trajectory also makes the path dependence clear. In the simulated SP–CH_3_ cycle, the zero-force optimization converged to an MC minimum; subsequent compression restored the closed SP structure. Within this optimization protocol, this behavior can reasonably be described as hysteresis-like or state-retaining, because the zero-force structure depends on its loading history, although it is not a conventional mechanical hysteresis loop. Figure 2 and Figure 3 define the approximate boundaries of the productive mechanochemical region. Under stretching, SP → MC first appears at approximately 0.040–0.045 a.u. (3.30–3.71 nN), while structural degradation is observed around 0.07–0.08 a.u. (5.77–6.59 nN). Under compression, the corrected scan places MC → SP closure near 0.026 a.u. (2.14 nN), whereas destructive changes again appear only near approximately 0.07 a.u. (5.77 nN). Figure 4 then probes two points—0.050 and 0.065 a.u. (4.12 and 5.36 nN)—inside these non-destructive ranges and shows that the complete switching cycle remains accessible at both. Such a productive regime is central to polymer mechanochemistry. A useful mechanophore must respond before nonspecific covalent scission or macromolecular damage becomes dominant [1,2]. Spiropyran is especially attractive in this respect because mechanically induced C–O opening creates a strongly detectable MC state.

Davis et al. demonstrated this principle in stress-responsive polymeric materials, where SP activation reports mechanical deformation before macroscopic failure [1]. Absolute EGO forces, however, should not be compared directly with force-spectroscopy values. Gossweiler et al. found that spiropyran ring opening on a tens-of-milliseconds timescale occurs at approximately 240 pN [6], whereas the present EGO transitions lie in the nanonewton range. This difference is expected because single-molecule force spectroscopy probes a thermally activated, loading-rate-dependent process [6,18], whereas EGO follows optimized structures under prescribed static external forces [19,20]. Calculated limiting forces for covalent-bond deformation or rupture are also sensitive to the computational definition and electronic-structure method used [26,30]. EGO forces are therefore most useful comparatively: they show where structural transitions and degradation occur within one internally consistent computational protocol, rather than predicting experimental rupture forces.

The substituent study considered below reinforces this point. Single-molecule experiments show that electron-withdrawing substitution can lower the force required to reach a given SP ring-opening rate [7]. Combined theoretical and experimental work also shows that substitution and the geometry of polymer attachment both control mechanochemical activation [8]. In the present derivative series, the first open-MC structures span 0.055–0.065 a.u. (4.53–5.36 nN), indicating that chemical substitution can shift the lower boundary of productive activation. These data do not show how the upper degradation boundary changes; establishing that would require dedicated high-force degradation scans for every derivative.

Figure 4 should therefore be read as one complete calculated switching cycle, not as evidence for unlimited cyclability. Repeated switching of spiropyran systems can be influenced by photochemical, thermal, and environmental processes [5]. The conclusion is more specific. For the investigated SP–CH_3_ model and loading geometry, a non-destructive force domain exists in which mechanical loading produces MC and reverse loading restores the original SP minimum. Both the position and width of this domain depend on the magnitude and direction of the applied force, and they are expected to change with substitution, force-bearing connectivity, and environment.

### 2.2. Substituent Effects on Force-Induced Spiropyran Ring Opening

**Figure 5:**
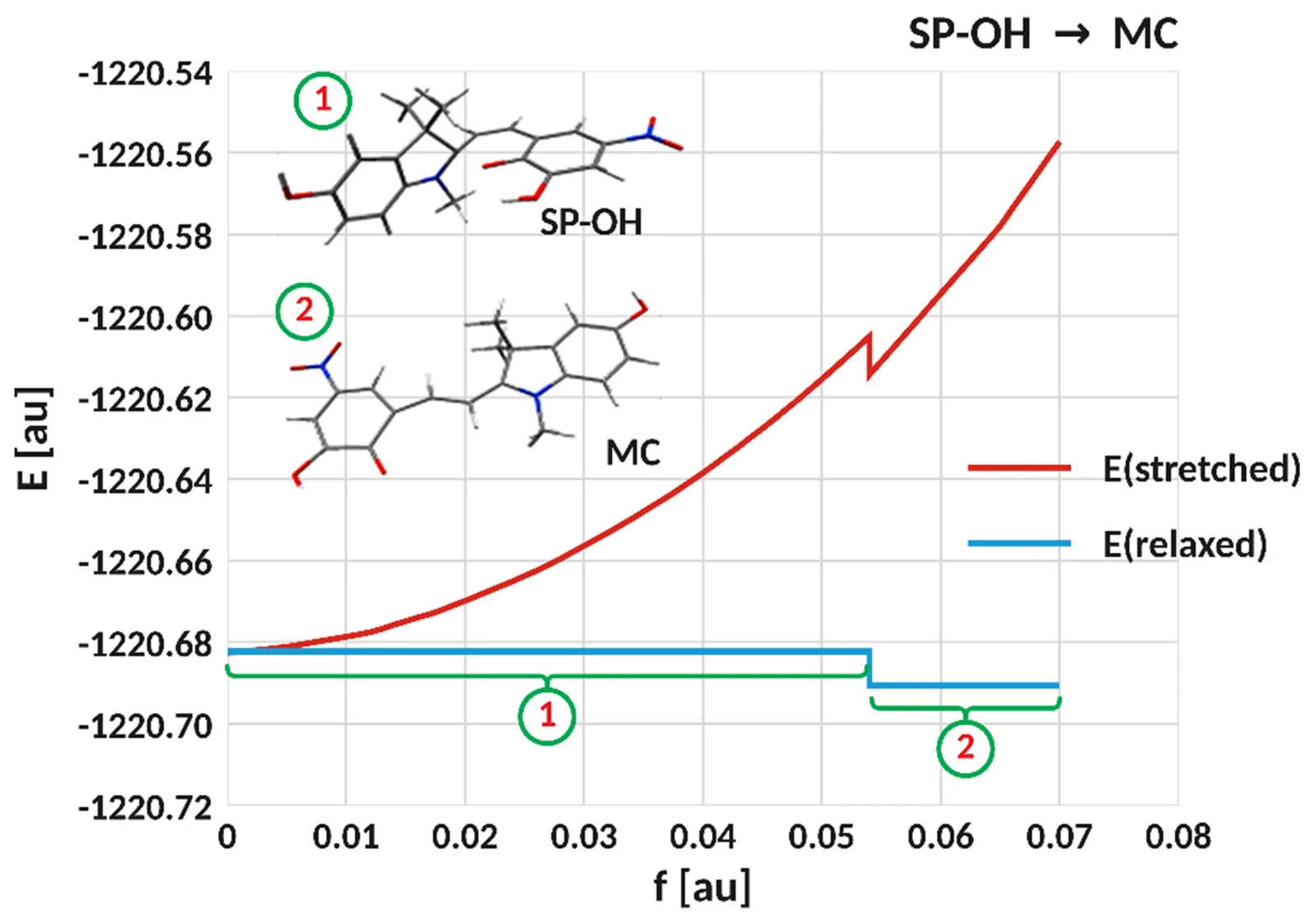
Molecular-energy profiles of the SP-OH model during tensile loading and subsequent zero-force relaxation. Force increment: 0.001 a.u. (0.082 nN). The coincident steps near 0.054-0.055 a.u. mark the SP → MC transition. E(relaxed) denotes energies after unloading; the horizontal coordinate identifies the preceding tensile load. The profiles include the additional calculations from f = 0 to 0.010 a.u. Plotted electronic energies exclude the external-force work term and are not activation barriers.

**Figure 6:**
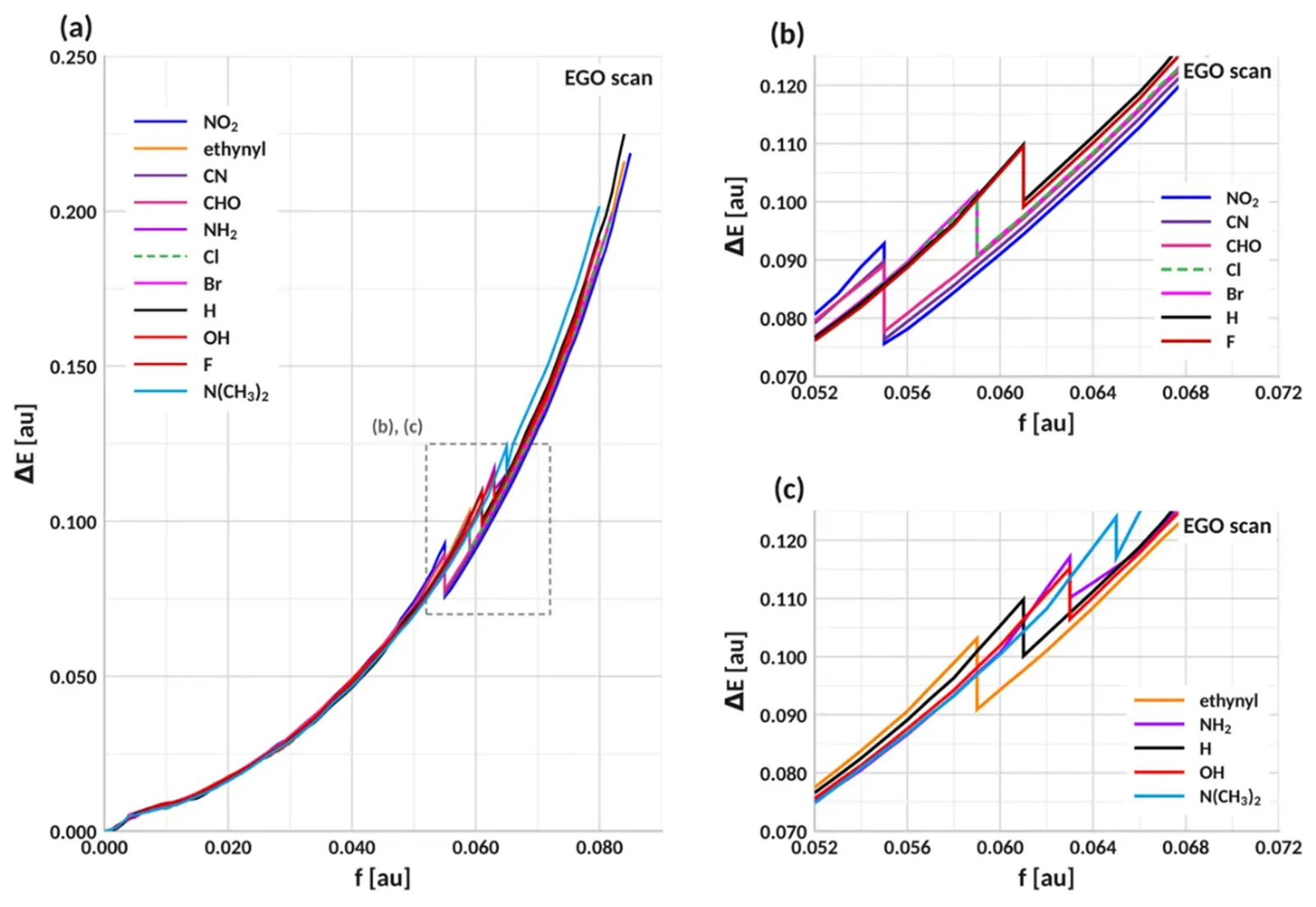
Relative-energy profiles from EGO stretching of substituted spiropyrans: (**a**) all derivatives, (**b**) enlarged ring-opening region for Z=NO_2_-, CN-, CHO-, Cl-, Br-, H-, and F-substituted systems, and (**c**) enlarged ring-opening region for Z=C≡CH- (ethynyl-), NH_2_-, H-, OH-, and N(CH_3_)_2_-substituted systems. The force increment is 0.001 a.u. (0.082 nN). For every derivative, ΔE(f) = E(f) − E(0), so ΔE(0) = 0. Plotted electronic energies exclude the external-force work term and are not activation barriers.

The SP–OH model gives an initial indication that even relatively small structural changes can alter the mechanical response of spiropyran. The recalculated SP–OH scan (X = Y = OH, Z = NO_2_; Figure 5) shows simultaneous discontinuous decreases in E(stretched) and E(relaxed) near 0.054–0.055 a.u. (4.45–4.53 nN), marking access to the open-MC branch. After removal of the force, this MC branch lies below the closed-SP branch. For the scans compared here, SP–OH opens at a higher tensile force than SP–CH_3_, whose transition occurs near 0.040–0.045 a.u. (3.30–3.71 nN). The revised opening region agrees, within the resolution of the plots, with the approximately 0.055 a.u. transition of the NO_2_-substituted derivative in Figure 6b. In both models, tensile loading first deforms closed SP and then cleaves the C_spiro_–O bond to generate an open MC-like product. This comparison does not isolate a purely electronic substituent effect, because changes in the molecular framework may also alter force transmission. Rather, it motivates a systematic analysis based on a common molecular framework and a common loading protocol.

Inspection of the existing zero-force H-, NH_2_-, Cl- and NO_2_-substituted structures supports an intramolecular O–H···O interaction between the adjacent hydroxy group and the spiro/phenolate oxygen. The contact is already present in closed SP and becomes shorter after opening and relaxation; available NBO results additionally show LP(O) → σ*(O–H) donation. This interaction can contribute to stabilization of the relaxed hydroxy-substituted MC structures, but the present comparison does not isolate its energetic contribution from other changes between SP–OH and SP–CH_3_ (Supporting Information, Section S11, Table S12).

In this SP–OH model, the forces act on the oxygen atoms of the two hydroxy groups; the output atom labels are given in the Supporting Information (Section S11).

Substitution is known to affect both SP–MC energetics and mechanochemical reactivity. Sheng et al. showed theoretically that substituents modify the SP–MC energy balance and conversion pathway [12], while Brügner et al. related SP–MC energy differences to Hammett parameters across a broader series of derivatives [31]. Under mechanical loading, Barbee et al. found experimentally that electron-withdrawing substitution can reduce the force required for ring opening [7]. Connectivity and substitution position matter as well: Gossweiler et al. and Kempe et al. both showed that these features influence activation [6,8]. Substituent effects in spiropyran mechanochemistry should therefore reflect not only electronics, but also how efficiently force is transmitted through the molecular framework.

To examine this question systematically, eleven derivatives were studied within the common SP–OH framework (fixed X = Y = OH), with Z varied across NO_2_, CN, CHO, Cl, Br, C≡CH, F, H, NH_2_, OH, or N(CH_3_)_2_. The set spans electron-withdrawing, weakly perturbing, and electron-donating substituents and allows a direct test of whether the first observed SP → MC opening force follows a simple electronic trend. Figure 6a presents the complete EGO stretching profiles; Figure 6b,c enlarge the ring-opening region. For each derivative, the transition is bracketed by the last closed-SP geometry and the first clearly open-MC geometry. Because the scans use finite force increments, these values locate the observed structural transition but do not define an exact mechanical-instability threshold.

At low force, the full profiles are closely similar. The substituent-dependent differences become much clearer only as each structure approaches its ring-opening region. After C_spiro_–O cleavage, the profiles again follow broadly similar rising trends, although derivative-specific offsets and local features remain. In other words, the strongest discrimination between substituents occurs as the system leaves the closed-SP basin, not during the initial low-force deformation. The approximate positions of the opening steps are 0.055 a.u. for NO_2_, CN and CHO; 0.059 a.u. for Cl, Br and ethynyl; 0.061 a.u. for H and F; 0.063 a.u. for NH_2_ and OH; and 0.065 a.u. for N(CH_3_)_2_. This ordering shows a clear overall Hammett-like electronic trend: strongly electron-withdrawing substituents open earlier, whereas strongly electron-donating substituents open later. The finite force increment does not resolve a finer order within groups whose opening steps coincide. This agrees with earlier work showing that substituents alter SP–MC energetics [12,31], while the mechanochemical response also depends on force transmission and molecular connectivity [6,8].

The H, NH_2_, and Cl members of the series in Figure 6b,c were chosen for a more detailed electronic analysis. They occupy three distinct ring-opening intervals, which makes them a useful focused comparison, although they are not intended to represent the behavior of all eleven derivatives. At the common force used for this analysis, f = 0.065 a.u. (5.36 nN), all three systems are open. Table 1 summarizes the structural, electronic, and energetic descriptors for the closed, force-opened, and zero-force-relaxed structures. Differences in the mechanically active C_spiro_–O interaction are already visible before force is applied. NH_2_ has the shortest C_spiro_–O bond and the largest Wiberg bond index (WBI). Cl, in contrast, has the longest bond, the largest σ*(C_spiro_–O) occupancy, and the strongest LP(Nindoline) → σ*(C_spiro_–O) interaction. The WBI is highest for NH_2_ (0.8784), consistent with its shorter C_spiro_–O distance and higher opening-force interval. H and Cl, however, have nearly identical WBI values, 0.8495 and 0.8514, despite responding differently to force. The lower opening interval of Cl is instead accompanied by a somewhat longer C_spiro_–O bond, greater σ* occupancy, and stronger donor–acceptor delocalization. No single ground-state descriptor therefore reproduces the full mechanical sequence. The substituent effect appears to reflect coupled changes in local bonding, electronic delocalization, molecular geometry, and force transmission. This interpretation is consistent with the mechanochemical substituent effects reported experimentally by Barbee et al. [7] and with the combined substituent/connectivity dependence described by Kempe et al. [8].

At the common applied force, the covalent C_spiro_–O interaction is essentially absent in all three derivatives. The C_spiro_···O separations exceed 5.3 Å and the corresponding WBI values fall to approximately 0.02 (Table 1). The localized C_spiro_–O bonding NBO disappears as well. Together, these observations confirm cleavage of the spiro bond.

The electronic reorganization extends beyond bond cleavage. Ring opening produces π bonding in the extended framework [32], so the resulting structures are better regarded as electronically reorganized MC-like species than as highly stretched closed spiropyrans. NBO analysis provides a localized-orbital view of this change [33,34]. The spatial delocalization of canonical frontier orbitals can complicate the identification of reactive regions. Yu et al. addressed this limitation by introducing frontier molecular orbitalets localized in both physical and energy spaces [35]. The HOMO–LUMO gaps also decrease substantially after opening in all three systems (Table 1). The largest reduction occurs for NH_2_, even though this derivative has the highest first-opening force of the three. The frontier-orbital gap of the post-cleavage product therefore does not directly predict the force required to leave the closed-SP basin. The electrostatic descriptors tell a related, but not identical, story. CHELP analysis shows pronounced charge redistribution, and the molecular dipole moment rises markedly in every derivative, reaching its largest value for Cl. A larger dipole moment for MC than for SP is consistent with computational studies [36,37] and experimental electrooptical measurements of spiropyran/merocyanine systems [38]. The oxygen CHELP charge and the total dipole moment do not, however, follow the same substituent order. In the force-opened state, for example, NH_2_ gives a more negative oxygen charge than Cl, whereas Cl has the larger overall dipole moment. The two descriptors therefore capture different aspects of the redistribution: the CHELP charge is local, while the dipole reflects the spatial charge distribution of the molecule as a whole.

ESP-fitted atomic charges depend on both molecular geometry and the fitting procedure [39]. For that reason, the coordinated change in molecular polarization is mechanistically more robust than the absolute charge assigned to a single atom. The structural and electronic descriptors nevertheless point in the same direction: a large Cspiro···O separation, near-zero WBI, loss of the localized Cspiro–O NBO, development of π conjugation, and a smaller HOMO–LUMO gap. Together, they provide a consistent electronic picture of SP → MC conversion, in agreement with previous computational and spectroscopic descriptions of SP/MC electronic structures [12,32,36,37].

The relative electronic energies at f = 0.065 a.u. (5.36 nN) distinguish the derivatives in another way. Relative to the respective optimized closed structures, E is +0.11491 a.u. (+301.70 kJ mol^−1^) for H, +0.10341 a.u. (+271.50 kJ mol^−1^) for NH_2_, and +0.11228 a.u. (+294.79 kJ mol^−1^) for Cl (Table 1). These numbers are not activation energies. Indeed, NH_2_ has the smallest E at the common post-opening force, yet it also has the highest first-opening force of the three derivatives. The energy of a post-cleavage structure at one selected force is therefore distinct from the force-dependent energetic conditions that govern departure from the closed-SP basin. Nor does removal of the external force restore the C_spiro_–O bond in any of the three systems. The relaxed structures retain large C_spiro_···O separations and WBI values close to zero, and the localized C_spiro_–O bonding NBO remains absent.

The conjugated open framework and the reduced HOMO–LUMO gaps persist after the force is removed. The relaxed products show small differences in relative electronic energy. With respect to the corresponding closed structures, ΔE_r_ is +0.00125 a.u. (+3.28 kJ mol^−1^) for H, +0.00326 a.u. (+8.55 kJ mol^−1^) for NH_2_, and −0.00067 a.u. (−1.77 kJ mol^−1^) for Cl (Table 1). These are electronic-energy differences, not Gibbs free energies, and they should not be interpreted as equilibrium populations. At B3LYP/6-311G(d,p), the order ΔE_r_(Cl) < ΔE_r_(H) < ΔE_r_(NH_2_) parallels the observed opening-force order. This endpoint association is descriptive: it does not establish the energetic origin of the opening-force differences or select an applied force. The small energy differences are specific to the level of theory and mechanically reached structures. This is consistent in a broad sense with earlier work showing substituent-dependent SP–MC energetics [12,31], but the H/NH_2_/Cl ordering found here is specific to these mechanically selected structures. Even so, the nearly identical closed-state WBI values of H and Cl show that bond order alone cannot account for the force-response sequence. Paired zero-force endpoint energies for all eleven derivatives are listed in Table S7. For NO_2_, CHO, CN, Br, F, C≡CH, OH and N(CH_3_)_2_, ΔE_r_ = −22.76, −3.56, −6.17, −1.75, −0.30, −1.78, −0.57 and −0.69 kJ mol^−1^, respectively.

Separate gas-phase checks assess the sensitivity of the small zero-force SP–MC energy differences (Supporting Information, Tables S8 and S9). Harmonic ZPE preserves their signs for H, NH_2_ and Cl, while diffuse basis functions preserve the signs for the tested NH_2_ and Cl pairs. These results concern the sampled conformers and do not establish equilibrium populations or opening barriers.

The complete force scans and the common-force electronic calculations therefore address two complementary aspects of substituent control. The EGO scans show when the optimized pathway first enters the open-MC basin. Table 1, by contrast, describes the electronic structure and relative energetics before cleavage, after opening under the same applied force, and after the force is removed. The picture that emerges is multicomponent: electronic preactivation contributes to the mechanochemical response, but no single descriptor universally predicts the observed EGO ring-opening interval.

A focused fixed-geometry D3(BJ) test was performed for existing H, NH_2_ and Cl optimization sequences, with force-modified potentials evaluated at a common nominal force of 0.065 a.u. (Supporting Information, Section S9.3, Table S10). Over the selected opening segments, the dispersion contribution increases by 8.85, 8.71 and 10.80 kJ mol^−1^, respectively, while the evaluated potential remains descending along the sampled geometries. This energy-sensitivity test does not determine dispersion-corrected opening forces or activation barriers; the nominal-force convention for NH_2_ is stated explicitly in the Supporting Information.

### 2.3. Solvent Effects on Force-Induced Spiropyran Ring Opening

Solvent effects were investigated for the SP–OH model (X = Y = OH, Z = NO_2_; Scheme 1), also examined in Figure 5. The role of the molecular environment was examined in the gas phase and with COSMO continuum descriptions of n-heptane, CCl_4_, toluene, o-xylene, diethyl ether, CHCl_3_, methyl benzoate (MB), aniline, THF, 1,2-dichloroethane (1,2-DCA), pyridine, DMF, and 1-butanol. Three structures were considered in each environment: optimized closed spiropyran (s), the force-generated structure obtained at the common force f = 0.065 a.u. (5.36 nN) (e), and the structure obtained after complete removal of the external force (r). Their structural descriptors and zero-force relative electronic energies ΔE_r_ are summarized in Table 2. The common-force comparison was kept separate from the first geometry-confirmed SP → MC points obtained in the individual EGO scans. Those points depend on the force increment and correspond to the first sampled optimized geometry that enters an open-MC region of the force-modified potential-energy surface. They are therefore neither kinetic activation forces nor exact thresholds of mechanical instability. This distinction matters when the calculations are compared with single-molecule force spectroscopy, in which the measured characteristic force depends on the loading conditions and force-dependent kinetics [6,18].

Even before mechanical loading, the optimized closed SP structures show a solvent dependence in the mechanically active C_spiro_–O bond. Relative to the gas phase, solvation generally gives a small bond elongation. At the same time, the bond order decreases, the population of the corresponding σ*(C_spiro_–O) orbital increases, the LP(Nindoline) → σ*(C_spiro_–O) donor–acceptor stabilization becomes somewhat stronger, and the HOMO–LUMO gap narrows.

These trends are consistent with previous theoretical studies showing that solvent can alter SP–MC energetics, charge distribution, molecular polarity, and the relative stabilization of the closed and open forms [12,36,37,40]. The full set of Wiberg bond-order, NBO, dipole-moment, and frontier-orbital descriptors is given in Table S5.

The individual descriptors do not follow a simple monotonic dependence on dielectric constant. That is not unexpected: the response of the SP/MC system cannot, in general, be reduced to bulk permittivity alone. Continuum models also omit explicit directional solute–solvent contacts, which can change the relative stabilization of closed and open structures [40,41].

**Figure 7:**
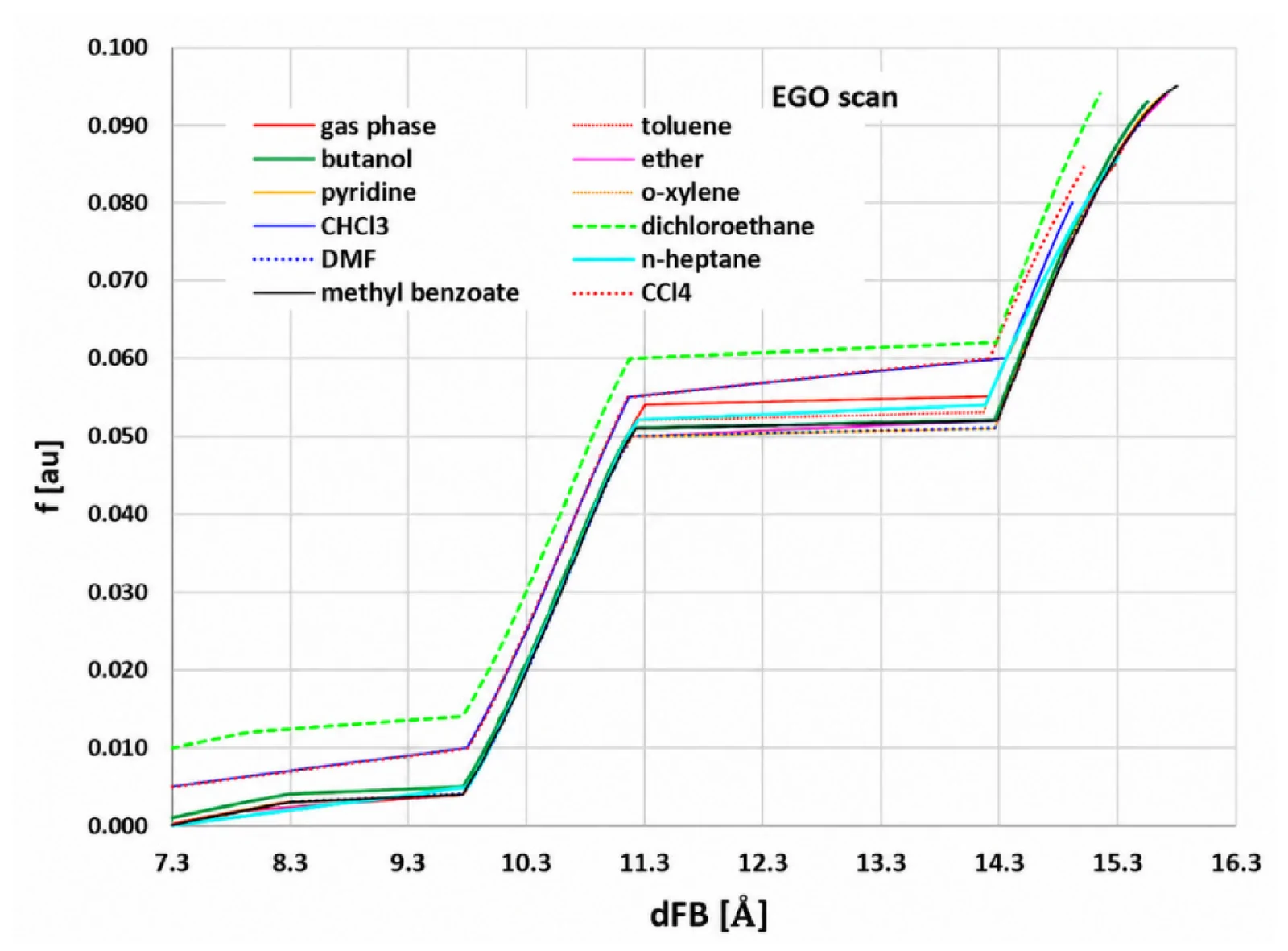
Selected EGO force–extension profiles for the SP–OH model (X = Y = OH, Z = NO_2_; Scheme 1) in the gas phase and in the investigated COSMO solvent environments. L denotes the force-bearing distance and f the applied external force.

Table 2 already shows a solvent effect in closed SP, although the changes in bond length are small. Much larger differences emerge after the force is removed, when the environments lead to distinct MC conformational families. Figure 7 compares the available complete force–extension profiles. The trajectories differ quantitatively, but they share the same broad pattern: a low-force structural accommodation is followed by the much larger extension associated with SP → MC conversion.

The additional ΔE_r_ descriptor spans −22.76 to +6.40 kJ mol^−1^ across the gas phase and thirteen continuum media (Table 2 and Table S6). At the reported level of theory, the mechanically reached relaxed MC structure lies below the corresponding closed SP in the gas phase, n-heptane, CCl_4_, toluene, aniline, 1-butanol and DMF, and above it in the other media. The values do not vary monotonically with dielectric constant. They compare the conformers reached by zero-force relaxation and do not establish equilibrium conformer populations or a general ordering of opening forces.

The detailed EGO scans help separate these two responses. The low-force feature involves a substantial change in overall molecular extension without elongation of the mechanically active C_spiro_–O bond. It is therefore assigned to intramolecular geometric reorganization of the closed SP framework, not to the onset of bond cleavage. Such preliminary conformational motion is compatible with the known flexibility and multidimensional character of the SP–MC potential-energy landscape [13,32]. Because the spiro C–O bond remains intact, however, the event observed here should not be identified directly with a previously proposed thermal, photochemical, or mechanochemical intermediate. The Supporting Information provides a detailed geometrical analysis of this low-force response, including nitrogen geometry, methyl-group torsions, and the force-bearing distance (Figures S1–S3 and Table S2). The event at higher force is structurally different. Here, the C_spiro_···O distance jumps from the covalent-bond region to values above 5 Å, while the emerging MC chain reorganizes substantially. This feature is therefore assigned to genuine SP → MC ring opening. The most conservative description of the calculated sequence is geometric reorganization → spiro C–O cleavage → MC conformational relaxation. At f = 0.065 a.u. (5.36 nN), every environment listed in Table 2 has already entered the open region. The C_spiro_···O separation ranges from approximately 5.40 to 5.85 Å, confirming complete disruption of the original spiro C–O interaction. Ring opening is also accompanied by substantial electronic redistribution, including a smaller HOMO–LUMO gap and greater molecular polarity. The corresponding electronic descriptors are reported in Table S5.

The calculated solvent response is qualitatively consistent with the single-molecule measurements of Lin et al. [18], who found that the characteristic force associated with spiropyran opening decreases substantially as solvent polarity increases. The two quantities are not directly equivalent. The experimental value is a kinetic observable measured under finite loading conditions, whereas an EGO point identifies an optimized geometry on a force-modified potential-energy surface. A quantitative comparison of the absolute forces would therefore be inappropriate.

Removing the external force does not lead all systems to the same MC geometry (Table 2). Instead, four conformational families appear: TTC, CTC, CCC, and CCT; their detailed torsional parameters and force-bearing distances are given in Table S1. The gas phase, n-heptane, and CCl_4_ relax to TTC structures, whereas toluene gives CTC. The compact CCC family is obtained for o-xylene, diethyl ether, CHCl_3_, and aniline. MB, THF, 1,2-DCA, pyridine, 1-butanol, and DMF instead yield the more extended CCT family. Several open conformations are therefore accessible, consistent with the established multiminimum character of the MC potential-energy surface [13,32]. Experimental measurements likewise support structurally distinct cisoid and transoid merocyanine populations [28]. The compact CCC structures deserve particular care in interpretation. After force removal, their C_spiro_···O distances decrease to approximately 2.74 Å, yet the corresponding bond orders remain very small. These are compact open MC structures, not reclosed spiropyrans. Overall molecular dimensions provide another useful structural fingerprint. The global molecular extension was characterized by d_FB_, defined as the distance between the two force-bearing atoms to which the external forces were applied. TTC remains extended, with d_FB_ ≈ 12.66–12.67 Å; CCC contracts to approximately 8.39–8.42 Å; and CCT remains more extended, with d_FB_ ≈ 12.13–12.19 Å. Including DMF extends the CCT series into a substantially more polar continuum environment, but the conformer assignment still does not map one-to-one onto dielectric constant. Aniline, for example, gives CCC, whereas THF, 1,2-dichloroethane, pyridine, 1-butanol, and DMF all give CCT. Solvent therefore affects both the electronic predisposition of closed SP toward mechanical activation and the region of the multidimensional MC landscape reached after cleavage. The relaxed structures should consequently be viewed as pathway-selected local minima rather than predictions of equilibrium conformer populations.

Taken as a whole, the solvent calculations show that solvation acts at several stages. It modifies the electronic state of closed SP before mechanical cleavage, influences the force-dependent opening trajectory, and affects the conformational basin reached after the ring opens. A low-force intramolecular geometric reorganization comes first; the genuine SP → MC conversion then produces much larger structural and electronic changes. Neither the first geometry-confirmed EGO opening point nor the final MC conformation is determined by dielectric constant alone.

## 3. Materials and Methods

The EGO and electronic-structure calculations were performed with PQS, version 4.1 [42]. DFT calculations used the B3LYP functional [43,44] and the 6-311G(d,p) basis set [45]. Molecular structures were first optimized without external force, and vibrational analysis was used to confirm the stability of the resulting geometries. Structural changes under mechanical load were then examined with the EGO method [19]. In its original formulation, EGO combines quantum-chemical geometry optimization with an external tensile or compressive force applied to arbitrarily selected atomic nuclei. The approach was first illustrated through force-induced conformational changes of pyranose rings and the generation of alternative azobenzene isomers [19].

Repeating the optimization while changing the applied force makes it possible to follow force-induced structural transformations along a mechanically selected pathway. Baker and Woliński later applied the method—explicitly termed EGO—to force-driven stilbene isomerization and showed that it can generate metastable structures and provide useful starting geometries for transition-state searches linking the initial and final structures [20]. In the present calculations, equal and oppositely directed external forces were applied to the selected force-bearing atoms using the geometry shown in Figure 1. Scheme 1 summarizes the molecular framework and substitution pattern of the spiropyrans studied here. The atom-numbering scheme used in the structural and electronic analyses is provided in the Supporting Information (Section S10, Figure S5).

For the SP–OH model with X = Y = OH and Z = NO_2_, the force-bearing atoms are the two hydroxyl oxygens, O43 and O44 in the output numbering (Supporting Information, Section S11).

The external force was increased in steps of 0.001 a.u. (0.082 nN) for the revised EGO force profiles. At each value, the molecular geometry was optimized and then used as the starting structure for the next calculation. Stretching scans were continued to approximately 0.08 a.u. (6.59 nN). The resulting geometries were inspected for cleavage of the spiro C–O bond and formation of an open MC framework.

Geometry optimization was accepted as converged when the maximum gradient component was below 3 × 10^−4^ a.u. (GTOL) and either the absolute energy change was below 1 × 10^−6^ E_h_ (ETOL) or the maximum predicted displacement was below 3 × 10^−4^ in the optimizer coordinate convention (DTOL), or both. SCF convergence required the maximum Brillouin matrix element to be below 1 × 10^−5^ a.u.; the final integral-screening threshold was 1 × 10^−10^. These criteria apply to optimization under the specified external force. In the high-force damage regime, structural disruption was accompanied by SCF nonconvergence, preventing further geometry optimization. Configurations reached in this region are reported as distorted geometries, not converged stationary points. The upper boundary is resolved only within the sampled force range. The term structural degradation describes damage observed along the imposed loading pathway and does not by itself establish whether the damaged structure would recover after unloading.

After stretching, the generated MC structure was reoptimized with the external force removed. Harmonic vibrational analyses were performed for the closed SP and zero-force-relaxed MC structures of all eleven gas-phase substituted derivatives and for the solvent-study model in the gas phase and all thirteen COSMO solvents. All internal vibrational frequencies were positive, confirming local-minimum character at the reported level of theory (Tables S6 and S7). These results do not establish the kinetic lifetimes of MC. The full tensile loading–relaxation–compression–relaxation cycle was studied only for the SP–CH_3_ model, with compression starting from its zero-force-relaxed MC geometry.

For part of the data set, molecular energy was expressed as the simple difference ΔE(f) = E(f) − E(0), where E(f) is the electronic energy of the geometry optimized under force f and E(0) is the energy of the corresponding initial, zero-force structure. For the relaxed open structures, ΔE_r_ = E(MC_r_, f = 0) − E(SP_s_, f = 0), with the corresponding closed SP in the same medium as the reference. Energy differences were converted using 1 E_h_ = 2625.49963948 kJ mol^−1^. For COSMO calculations, the PQS total energy including the dielectric contribution was used consistently; the separately printed “Total energy + OC corr.” was not used. These values contain no zero-point or thermal correction and are not Gibbs free energies or activation barriers. Natural Bond Orbital (NBO) populations were analyzed within the NBO framework [33,34]. Wiberg bond indices (WBI), calculated with PQS, were used consistently to monitor changes in the effective C_spiro_–O bond order. CHELP electrostatic potential charges and molecular dipole moments derived from the charge distribution were obtained with the electrostatic-potential fitting procedure developed by Chirlian and Francl [39]. HOMO–LUMO gaps are reported as differences between Kohn–Sham orbital energies. They are used only as descriptors of changes in electronic structure, not as excitation energies or mechanical activation barriers. Solvent effects were modeled with the conductor-like screening model (COSMO) framework [46].

For a constant tensile force, the force-modified potential is V(R; f) = E(R) − fL(R), where L is the distance between the force-bearing atoms, expressed in bohr when f is in atomic units. The molecular electronic-energy profiles in Figure 2, Figure 3, Figure 4, Figure 5 and Figure 6 exclude this external-force term. Neither their maxima nor intermediate optimizer geometries are identified as transition states. Transition states and activation barriers at zero or finite force were not determined. Quantitative comparison with force-dependent kinetics [7] would require identifying the relevant conformational pathways and validating transition structures and their connections at the corresponding applied forces, beyond the scope of the present structural investigation.

Separate zero-force energy-sensitivity tests were performed for the gas-phase H-, NH_2_- and Cl-substituted systems. Unscaled harmonic ZPE values were taken from their B3LYP/6-311G(d,p) frequency analyses. For NH_2_ and Cl, B3LYP/6-311+G(d,p) single-point energies were evaluated on the B3LYP/6-311G(d,p) geometries. These tests are reported separately in Tables S8 and S9; they were not combined with the dispersion correction.

Pairwise D3(BJ) energies were evaluated with simple-dftd3 1.6.0 on 96 H, 203 NH_2_ and 144 Cl geometries from existing B3LYP/6-311G(d,p) optimization outputs. Standard B3LYP parameters were used: s_6_ = 1, s_8_ = 1.9889, a_1_ = 0.3981 and a_2_ = 4.4211; the ATM term was omitted. The potential V = E(B3LYP) + E(D3) − fL was evaluated at a nominal force f = 0.065 a.u. For NH_2_, this is an assumed energy-evaluation parameter; the printed source-force norm is 0.060 a.u. No dispersion-corrected optimization, ZPE, or thermal corrections were included in this test. Full conventions and the selected geometry intervals are given in Section S9.3.

Electronic stability was examined in Gaussian 16 using B3LYP/6-311G(d,p), Stable=Opt, SCF=(Tight,XQC), an UltraFine integration grid and NoSymm on the fixed PQS geometries listed in Table S11: the last closed and first open structures of H (source f = 0.060 and 0.062 a.u.), NH_2_ (0.064 and 0.066 a.u.) and Cl (0.058 and 0.060 a.u.). The initial restricted solution was stable under the perturbations considered in all six tests. These checks concern the reported geometries and do not establish stability for every point of each EGO path.

## 4. Conclusions

The EGO calculations provide a consistent molecular picture of mechanically driven SP–MC switching. In the SP–CH_3_ model, tensile loading cleaves the spiro C–O bond. The relaxed MC product is a local minimum at zero force, while compression restores the closed, initial SP form (the energy minimum state). Across eleven substituted spiropyrans, the SP → MC opening region spans approximately 0.055–0.065 a.u. (4.53–5.36 nN) and shows a clear overall Hammett-like trend, with strongly electron-withdrawing substituents opening earlier and strongly electron-donating substituents opening later. The finite force increment limits distinctions between derivatives whose opening steps coincide. The detailed H, NH_2_, and Cl comparison further shows that bond order, NBO analysis, electrostatic interactions, and frontier-orbital characteristics each describe part of the response, but none alone predicts how the system responds to mechanical force.

The solvent calculations add another layer to this picture. A geometric reorganization occurs at low force before bond rupture, and the final product reached after stretching depends on the solvent, giving TTC, CTC, CCC, and CCT MC conformers. These effects are not controlled by dielectric constant alone. The calculated force values should therefore be treated as pathway-specific parameters of the EGO model, useful for comparing responses within the same computational framework rather than as experimental kinetic activation forces. The focused dispersion test shows an energetic penalty over the sampled opening segments without reversing the evaluated potential decrease at the chosen nominal force. The opening intervals remain results of the stated B3LYP/EGO protocol; their changes after dispersion-corrected optimization, activation barriers and MC kinetic lifetimes have not been determined. Comparison with force-spectroscopy substituent trends is qualitative.

## Figures and Tables

**Table 1:** Structural, electrostatic, and electronic descriptors of the H-, NH_2_-, and Cl-substituted spiropyrans in the gas phase; r(Cspiro···O) is the distance between the former spiro carbon and oxygen atoms; WBI is the corresponding Wiberg bond index; qCHELP(O) is the CHELP electrostatic-potential charge on the oxygen atom formerly involved in the spiro C–O bond; μ is the molecular dipole moment. ΔE denotes the electronic-energy difference from the corresponding closed SP structure; for state r, ΔE_r_ = E(MC_r_, f = 0) − E(SP_s_, f = 0). Additional endpoint energy data are given in Table S7. These electronic-energy differences are not activation energies.

Z	State	r(C_spiro_···O), Å	WBI(C_spiro–_O)	q_CHELP(O),_ e	μ_CHELP_, D	HOMO–LUMO, eV	ΔE, a.u. (kJ mol^−1^)
H	*s*	1.4797	0.8495	−0.0274	2.562	4.023	0.00000 (0.00)
	*e*	5.3788	0.0188	−0.4573	7.558	2.397	+0.11491 (+301.70)
	*r*	4.1614	0.0184	−0.4779	7.772	2.458	+0.00125 (+3.28)
NH_2_	*s*	1.4698	0.8784	+0.0115	2.645	3.969	0.00000 (0.00)
	*e*	5.4161	0.0207	−0.5233	5.758	2.183	+0.10341 (+271.50)
	*r*	4.1382	0.0224	−0.5728	5.679	2.207	+0.00326 (+8.55)
Cl	*s*	1.4823	0.8514	+0.0727	3.878	3.919	0.00000 (0.00)
	*e*	5.3784	0.0188	−0.4893	8.416	2.348	+0.11228 (+294.79)
	*r*	4.1650	0.0185	−0.5875	8.659	2.433	−0.00067 (−1.77)

*s*—optimized closed SP structure at f = 0; *e*—force-opened structure; *r*—structure obtained after zero-force relaxation.

**Table 2:** Selected solvent-dependent structural descriptors and relative electronic energies of the optimized closed SP structure (s), the force-opened structure obtained at f = 0.065 a.u. (5.36 nN) (e), and the structure obtained after removal of the external force (r). ΔE_r_ = E(MC_r_, f = 0) − E(SP_s_, f = 0), evaluated separately in each medium. Absolute energies are listed in Table S6.

Medium	ε	r_s_ C_spiro_-O, Å	r_e_ C_spiro_⋅⋅⋅O, Å	r_r_ C_spiro_⋅⋅⋅O, Å	Relaxed MC	ΔE_r_, kJ mol^−1^
gas phase	—	1.4974	5.3983	4.1723	TTC	−22.76
n-heptane	1.920	1.5011	5.4009	4.1862	TTC	−18.52
CCl_4_	2.228	1.5024	5.4026	4.1902	TTC	−20.68
toluene	2.379	1.5047	5.5287	4.1684	CTC	−13.80
o-xylene	2.570	1.5021	5.8101	2.7363	CCC	+6.40
diethyl ether	4.335	1.5059	5.7965	2.7375	CCC	+2.40
CHCl_3_	4.900	1.5066	5.8022	2.7385	CCC	+1.36
MB	6.600	1.5061	5.8501	5.2962	CCT	+5.64
aniline	6.890	1.5081	5.8226	2.7407	CCC	−1.11
THF	7.580	1.5086	5.8496	5.2979	CCT	+4.82
1,2-DCA	10.360	1.5092	5.8501	5.2948	CCT	+2.63
pyridine	12.300	1.5081	5.8514	5.2891	CCT	+1.24
1-butanol	17.100	1.5085	5.8522	5.2874	CCT	−0.37
DMF	36.700	1.5095	5.8540	5.2860	CCT	−2.68

## Data Availability

Dataset is available upon request from the authors.

## Supplementary Materials

The following supporting information can be downloaded at: https://www.mdpi.com/article/10.3390/ijms27219767/s1.

## Abbreviations

The following abbreviations are used in this manuscript:

SP	Spiropyran.
MC	Merocyanine.
EGO	Enforced Geometry Optimization.
CHELP	CHarges from ELectrostatic Potentials.
