# Supplementary material for: Force-Induced Spiropyran–Merocyanine Interconversion: An Enforced Geometry Optimization Study
Source: Int J Mol Sci. 2026 Oct 30;27(21):9767. doi: 10.3390/ijms27219767 (PMC13763355; doi:10.3390/ijms27219767)
Supplement: Supplementary file 1 [file ijms-27-09767-s001.zip › ijms-4648044-supplementary.pdf]

Supporting Information

Force-induced spiropyran–merocyanine interconversion: an enforced geometry optimization study

Agnieszka Brzyska

S1. Definition and classification of relaxed MC conformers

The relaxed merocyanine structures were characterized using three consecutive torsional angles:

α = N12–C16–C28–C39 (atom numbering: Figure S5, Section S10)
β = C16–C28–C39–C31
γ = C28–C39–C31–C33
The three-letter conformer notation corresponds sequentially to the cis/trans character of α/β/γ.

Table S1. Geometric descriptors of relaxed MC structures.

Environment	ε	$r_r(C_{spiro}\cdots O)$, Å	d_{FB}, Å	α, °	β, °	γ, °	MC
Gas phase	—	4.1723	12.6600	−179.92	179.91	0.01	TTC
n-Heptane	1.920	4.1862	12.6706	179.92	179.88	−0.07	TTC
CCl_4	2.228	4.1902	12.6738	179.92	179.93	−0.13	TTC
Toluene	2.379	4.1684	12.3832	12.51	178.98	1.58	CTC
o-Xylene	2.570	2.7363	8.4236	−39.86	−17.49	−16.58	CCC
Diethyl ether	4.335	2.7375	8.4006	−41.25	−16.44	−17.03	CCC
$CHCl_3$	4.900	2.7385	8.3949	−41.53	−16.14	−17.15	CCC
Methyl benzoate	6.600	5.2962	12.1829	34.69	17.84	−171.76	CCT
Aniline	6.890	2.7407	8.3902	−42.40	−15.46	−17.28	CCC
THF	7.580	5.2979	12.1851	34.88	17.57	−171.80	CCT
1,2-Dichloroethane	10.360	5.2948	12.1679	35.61	16.97	−171.16	CCT
Pyridine	12.300	5.2891	12.1485	36.14	16.50	−170.25	CCT
1-Butanol	17.100	5.2874	12.1414	36.62	16.03	−169.83	CCT
DMF	36.7	5.2860	12.1261	−37.36	−15.46	169.28	CCT

The CCC and CCT families form compact clusters in torsional coordinates and global molecular dimensions. The signs of individual torsions reflect the adopted dihedral convention; conformer classification is based on the corresponding angular region rather than on sign alone.

S2. Detailed analysis of the low-force intramolecular geometric rearrangement

Complete EGO trajectories available for the gas phase, CCl_4, aniline, and 1-butanol were examined in detail to identify the structural origin of the first low-force response.

The analysis included the force-bearing distance L, the Cspiro–O distance, torsional coordinates associated with the methyl-bearing fragments, the N–CH_3 torsion, the signed displacement of N12 from the C5–C16–C25 plane, and the sum of the three valence angles around N12.

For Figures S1/S2 and Table S2, the ordered torsions are CH_3-A = C16–C13–C19–H20, CH_3-B = C16–C13–C22–H23, and N–CH_3 = C5–N12–C25–H26. A torsion A–B–C–D is the signed dihedral between planes A–B–C and B–C–D about bond B–C, using this fixed atom order. Atom indices follow the PQS outputs and Figure S5; hydrogen labels remain valid although hydrogen atoms are omitted from that drawing. The plotted angles are unwrapped by adding or subtracting 360° to maintain continuity between successive samples; 182° and −178°, for example, describe the same orientation.

The first force–extension discontinuity is not accompanied by elongation of the spiro C–O bond. Instead, the bond remains within the covalent range and in several trajectories becomes slightly shorter.

The most diagnostic structural change occurs at N12. In all four trajectories, the signed displacement of N12 relative to the plane defined by C5, C16, and C25 reverses sign across the low-force event. Under a fixed atom ordering, this sign reversal demonstrates passage of the nitrogen center through the substituent plane.

Immediately before the event, N12 approaches a near-planar geometry, particularly in the gas phase, aniline, and 1-butanol. After the discontinuity, the nitrogen becomes pyramidal again on the opposite side of the plane.

Npyramidal → Nnear-planar → Ninverted pyramidal

The inversion is accompanied by reorientation of the methyl-bearing framework. The internal N–CH_3 torsion itself changes only modestly, showing that the event should not be described primarily as free rotation around the N–C bond.

Table S2. Geometric changes bracketing the first low-force intramolecular rearrangement.

Environment	f before, a.u.	f after, a.u.	dN-plane before, Å	dN-plane after, Å	Σ∠N before, °	Σ∠N after, °	N–CH_3 before, °	N–CH_3 after, °
Gas phase	0.003	0.004	+0.195	−0.304	354.5	346.9	56.3	59.2
CCl_4	0.004	0.005	+0.276	−0.309	349.1	346.6	60.2	59.2
Aniline	0.003	0.004	+0.179	−0.306	355.3	346.8	55.8	59.6
1-Butanol	0.003	0.004	+0.178	−0.309	355.4	346.5	54.4	59.5

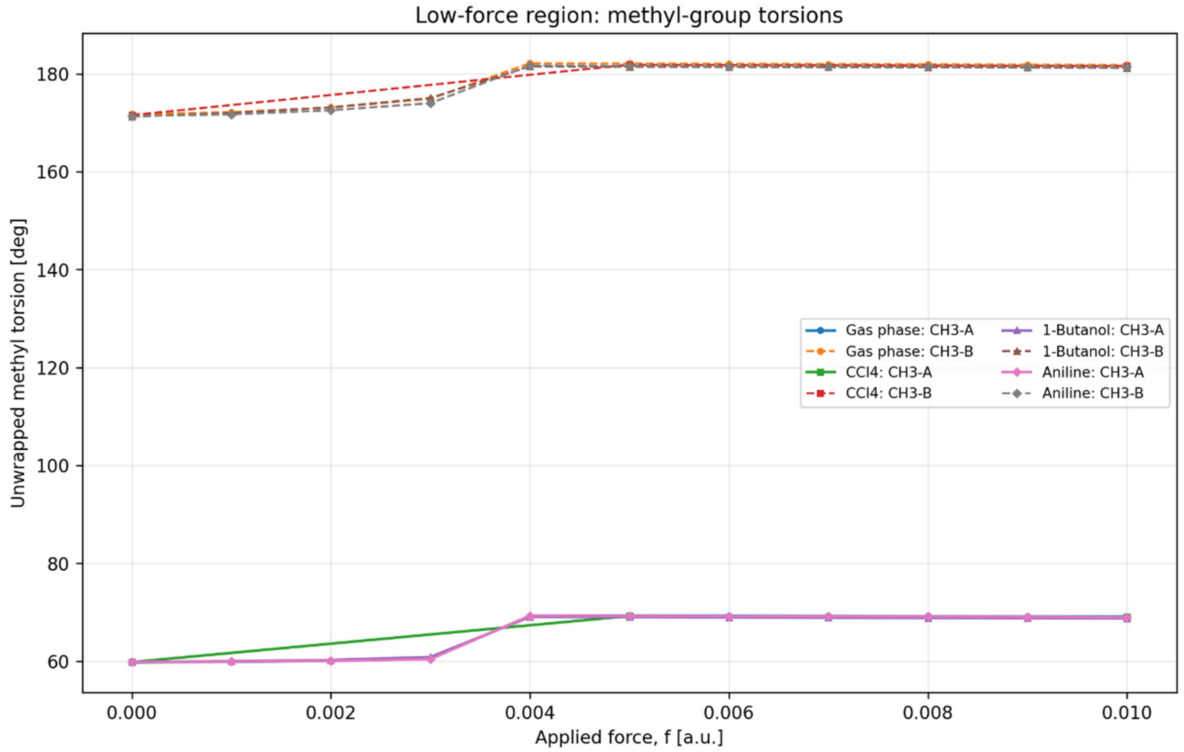

Figure S1. Low-force variation of torsional coordinates associated with the methyl-bearing fragments in the gas phase, CCl_4, aniline, and 1-butanol.

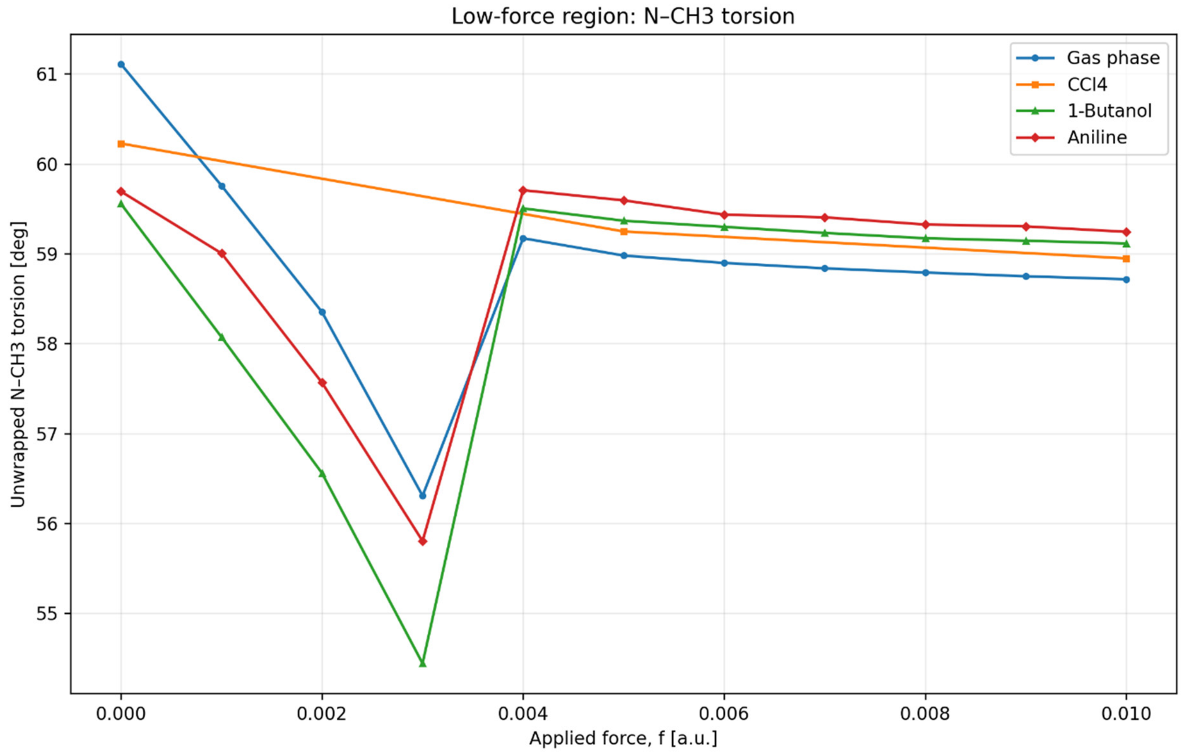

Figure S2. Variation of the N–CH_3 torsional coordinate in the low-force region. The relatively small torsional change compared with the inversion coordinate shows that the first structural response is not a simple rotation of the N-methyl group.

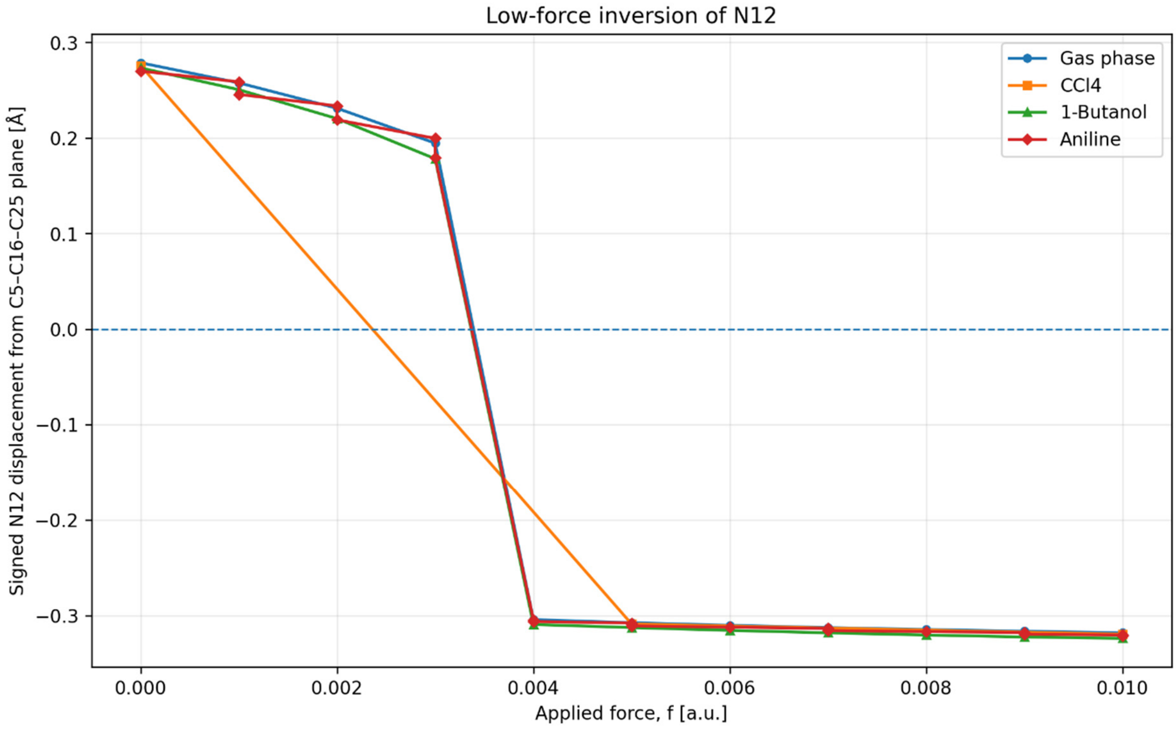

Figure S3. Signed displacement of N12 from the plane defined by C5, C16, and C25 as a function of applied force. The sign reversal associated with the first low-force discontinuity provides a geometric signature of pyramidal inversion of the indoline nitrogen.

Taken together, the geometric descriptors assign the first low-force feature to a concerted intramolecular rearrangement dominated by N12 inversion and reorientation of the surrounding framework, while the spiro C–O bond remains intact. The later SP→MC opening is characterized separately in Table S3.

S3. Geometry-confirmed SP→MC opening

The second mechanical event was identified directly from the molecular geometry rather than from the force–extension profile alone. The primary diagnostic criterion is abrupt elongation of the Cspiro···O distance from the normal covalent range to values above 5 Å, accompanied by substantial torsional reorganization of the developing MC chain.

Table S3. First open structures on the reported 0.001 a.u. force grid.

Environment	First open point, a.u.	L, Å	r(Cspiro···O), Å
Gas phase	0.055	14.2115	5.2282
CCl_4	0.055	14.2132	5.2345
Aniline	0.052	14.2833	5.7473
1-Butanol	0.051	14.2585	5.7488

For the broader solvent series, the scans give first geometry-confirmed open structures in approximately the 0.050–0.055 a.u. range. These values are useful for identifying pathway-dependent structural changes, but they are not experimental kinetic transition forces.

S4. NBO analysis of the N12 region

Natural population analysis was used to provide an electronic reference for the closed SP structure and the fully force-opened state. The NBO calculations presently available do not correspond to the two geometries immediately bracketing the first low-force rearrangement. Consequently, they cannot be used to quantify directly the electronic changes accompanying N12 inversion itself.

S4.1. Optimized closed SP structures

Table S4a. Natural charges and N12 lone-pair properties of optimized closed SP structures.

Environment	qNBO(N12), e	q(C25), e	q(C16spiro), e	q(O32), e	Occ. LP(N12), e	s, %	p, %
Gas phase	−0.49820	−0.35941	+0.49979	−0.59935	1.76561	5.83	94.15
CCl_4	−0.49731	−0.36286	+0.49825	−0.59582	1.76110	5.71	94.27
Aniline	−0.49587	−0.36550	+0.49714	−0.59256	1.75622	5.49	94.49
1-Butanol	−0.49750	−0.36729	+0.49701	−0.58998	1.75694	5.56	94.42
DMF	−0.49727	−0.36770	+0.49689	−0.58940	1.75619	5.53	94.45

The natural charge on N12 remains close to −0.50 e across the closed structures. The N12 lone pair is strongly p-type in every environment, with approximately 94% p character. Solvent-induced preactivation of the closed SP structure is therefore not expressed as a large change in the net natural charge localized on N12.

S4.2. Force-opened structures at f = 0.065 a.u.

Table S4b. Natural charges of force-opened structures at f = 0.065 a.u.

Environment	qNBO(N12), e	Δq(N12), e	q(C25), e	q(C16spiro), e	q(O32), e
Gas phase	−0.38866	+0.10954	−0.36076	+0.32988	−0.58374
CCl_4	−0.37392	+0.12339	−0.36518	+0.35054	−0.60881
Aniline	−0.35684	+0.13903	−0.35800	+0.36161	−0.62228
DMF	−0.34889	+0.14838	−0.35422	+0.37145	−0.66234

The difference between the closed and force-opened structures is much larger than the differences among the closed structures themselves. The force-opened structures show substantial charge redistribution at N12 and at the former spiro carbon and oxygen. These data clearly separate the modest solvent-dependent electronic differences of closed SP from the much larger electronic reorganization accompanying complete SP→MC opening.

The present endpoint data do not establish whether a smaller transient redistribution accompanies N12 inversion during the first low-force event. Direct characterization would require equivalent NBO calculations for geometries immediately before and after the inversion.

S5. Complete solvent-dependent electronic descriptors

The detailed electronic quantities omitted from Table 2 of the main manuscript are collected here.

Table S5. Complete solvent-dependent electronic descriptors of closed, force-opened, and relaxed structures.

Environment	WBI_s	n(σ*)_s	E(2)_s, Eh	Gap_s, eV	WBI_e	WBI_r	μ_e, D	μ_r, D	Gap_e, eV	Gap_r, eV
Gas phase	0.8166	0.10755	0.03049	3.245	0.0152	0.0140	12.878	13.469	2.330	2.460
n-Heptane	0.8115	0.11024	0.03111	3.085	0.0137	0.0119	15.257	15.715	2.342	2.512
CCl_4	0.8097	0.11105	0.03130	3.051	0.0133	0.0115	15.810	16.246	2.348	2.526
Toluene	0.8083	0.11274	0.03179	3.041	0.0129	0.0119	16.317	16.652	2.378	2.532
o-Xylene	0.8099	0.11065	0.03115	3.025	0.0114	0.0360	16.087	15.898	2.345	2.775
Diethyl ether	0.8043	0.11375	0.03197	2.915	0.0108	0.0353	17.641	17.217	2.334	2.828
$CHCl_3$	0.8030	0.11404	0.03203	2.896	0.0108	0.0349	18.435	17.529	2.320	2.840
Methyl benzoate	0.8040	0.11335	0.03174	2.868	0.0116	0.0105	16.967	16.603	2.434	2.558
Aniline	0.8009	0.11516	0.03229	2.848	0.0106	0.0346	20.360	18.246	2.269	2.869
THF	0.7997	0.11534	0.03237	2.836	0.0116	0.0104	17.059	16.777	2.433	2.561
1,2-Dichloroethane	0.7985	0.11588	0.03248	2.803	0.0112	<0.0100	17.770	17.275	2.438	2.584
Pyridine	0.8013	0.11468	0.03206	2.807	0.0107	<0.0100	18.304	17.613	2.448	2.607
1-Butanol	0.8004	0.11496	0.03211	2.784	0.0104	<0.0100	18.786	17.988	2.455	2.627
DMF	0.7989	0.11562	0.03227	2.753	<0.0100	<0.0100	19.660	18.687	2.467	2.657

Table S5 note. n(σ*) is the occupancy of the antibonding NBO associated with the mechanically active Cspiro–O bond. E(2) is the second-order LP(N)→σ*(C–O) stabilization energy, converted to Hartree using 1 Eh = 627.5095 kcal mol−1. The e and r labels denote the structure at f = 0.065 a.u. and the structure obtained after force removal, respectively.

DMF continues the overall closed-state trend observed for the more polar continuum environments. At the common force, the original spiro interaction has a bond order below the reporting threshold of 0.010, consistent with the large Cspiro···O separation.

S6. Structural fingerprints of the relaxed MC families

TTC: α ≈ ±180°, β ≈ 180°, γ ≈ 0°. These structures remain globally extended.

CTC: α ≈ 0°, β ≈ 180°, γ ≈ 0°. Toluene is the representative member of this group in the present solvent series.

CCC: α ≈ −40°, β ≈ −16°, γ ≈ −17°. These structures are strongly contracted globally, with dFB ≈ 8.4 Å.

CCT: |α| ≈ 35–37°, |β| ≈ 15–18°, |γ| ≈ 169–172°. These structures remain extended, with dFB ≈ 12.13–12.19 Å.

The inclusion of DMF strengthens the CCT assignment as a well-defined structural family. Despite its much larger dielectric constant, its relaxed global dimensions closely resemble those obtained for methyl benzoate, THF, 1,2-dichloroethane, pyridine, and 1-butanol. This finding further illustrates that the relaxed conformational basin is not determined by dielectric constant alone.

S7. Methodological considerations

The force values obtained from EGO scans identify optimized structures sampled along a prescribed mechanical trajectory. They are not kinetic activation forces and should not be interpreted as direct computational equivalents of characteristic forces measured in single-molecule force spectroscopy. In the substituent series (Figure 6 and Table S7), X = Y = OH is fixed and Z is varied. The gas-phase and thirteen-solvent series in Section 2.3 and Figure 7 of the main article uses the SP–OH model (X = Y = OH, Z = NO_2; Scheme 1), also examined in Figure 5.

The revised energy profiles in the main article used a force increment of 0.001 a.u. (0.082 nN). The focused dispersion comparison in Section S9.3 evaluates existing optimizer geometries at a nominal force and does not replace these force profiles. The first geometry-confirmed open structure therefore has a resolution determined by the sampling interval used for the corresponding trajectory. In the recalculated SP–OH scan, the coincident discontinuities in the loaded-state and zero-force-relaxed energies identify entry into the open-MC branch near 0.054–0.055 a.u.; their magnitude does not give an activation barrier.

In Figure 3, compression starts from a common zero-force MC reference and both energy profiles step near 0.026 a.u., marking MC→SP closure. In Figure 2, the steps near 0.043 and 0.064 a.u. distinguish entry into the cisoid-MC-like regime and access to transoid-MC-like structures; the relaxed MC branch remains above the initial SP energy.

In Figure 6, $\Delta E(f) = E(f) - E(0)$, with $\Delta E(0) = 0$ for all eleven series. The approximate positions of the opening steps are 0.055 a.u. for NO_2, CN and CHO; 0.059 a.u. for Cl, Br and ethynyl; 0.061 a.u. for H and F; 0.063 a.u. for NH_2 and OH; and 0.065 a.u. for $N(CH_3)_2$. This overall Hammett-like trend is consistent with earlier opening for stronger electron acceptors and later opening for stronger donors. The 0.001 a.u. step limits distinctions within coincident groups; the step positions are distinct from ΔE_r in Table S7.

COSMO represents bulk dielectric stabilization and does not explicitly include specific solute–solvent hydrogen bonding, local solvent orientation, or other individual solvent contacts. This limitation becomes increasingly relevant for strongly interacting or protic media.

The structures obtained after removal of the external force are local minima reached from mechanically generated geometries. Their occurrence does not imply that they represent equilibrium conformer populations in the corresponding bulk solvents.

Positive harmonic frequencies establish local-minimum character without determining activation barriers or MC lifetimes. This study concerns structural and electronic changes along EGO loading pathways. EGO minimizes $E - fL$; the plotted molecular energies omit the force term. Transition states and barriers at zero or finite force were not determined. A kinetic interpretation requires separately validated pathways connecting the relevant conformers.

The detailed analysis of the first low-force geometric rearrangement was performed for trajectories for which sufficiently resolved complete scans were available. Its microscopic assignment therefore applies specifically to the analyzed EGO pathways.

The available NBO endpoint calculations characterize closed SP and fully force-opened structures but do not directly sample the geometries immediately surrounding the low-force N12 inversion. The assignment of the first event therefore rests primarily on the direct geometric descriptors reported in Section S2.

The HOMO–LUMO gaps in Table S5 describe electronic reorganization during SP–MC conversion and are not used to infer reaction rates or mechanical activation forces. Spatial delocalization of canonical frontier orbitals can complicate the identification of reactive regions; Yu, Su and Yang (2022) addressed this limitation using frontier molecular orbitalets localized in both physical and energy spaces.

S8. Structural criterion for high-force degradation

At f = 0.070 a.u. (5.77 nN), the C34–C41 bond linking the force-bearing methyl group to the SP–CH_3 framework elongates from 1.80 to 3.89 Å (Figure S4). This damage, accompanied by SCF nonconvergence, occurs outside the intended spiro C16–O32 switching coordinate. The already-open C16···O32 separation increases from 5.59 to 6.08 Å. The affected bonds and the extent of damage depend on the molecular system; these distances describe this trajectory and do not define a general degradation threshold.

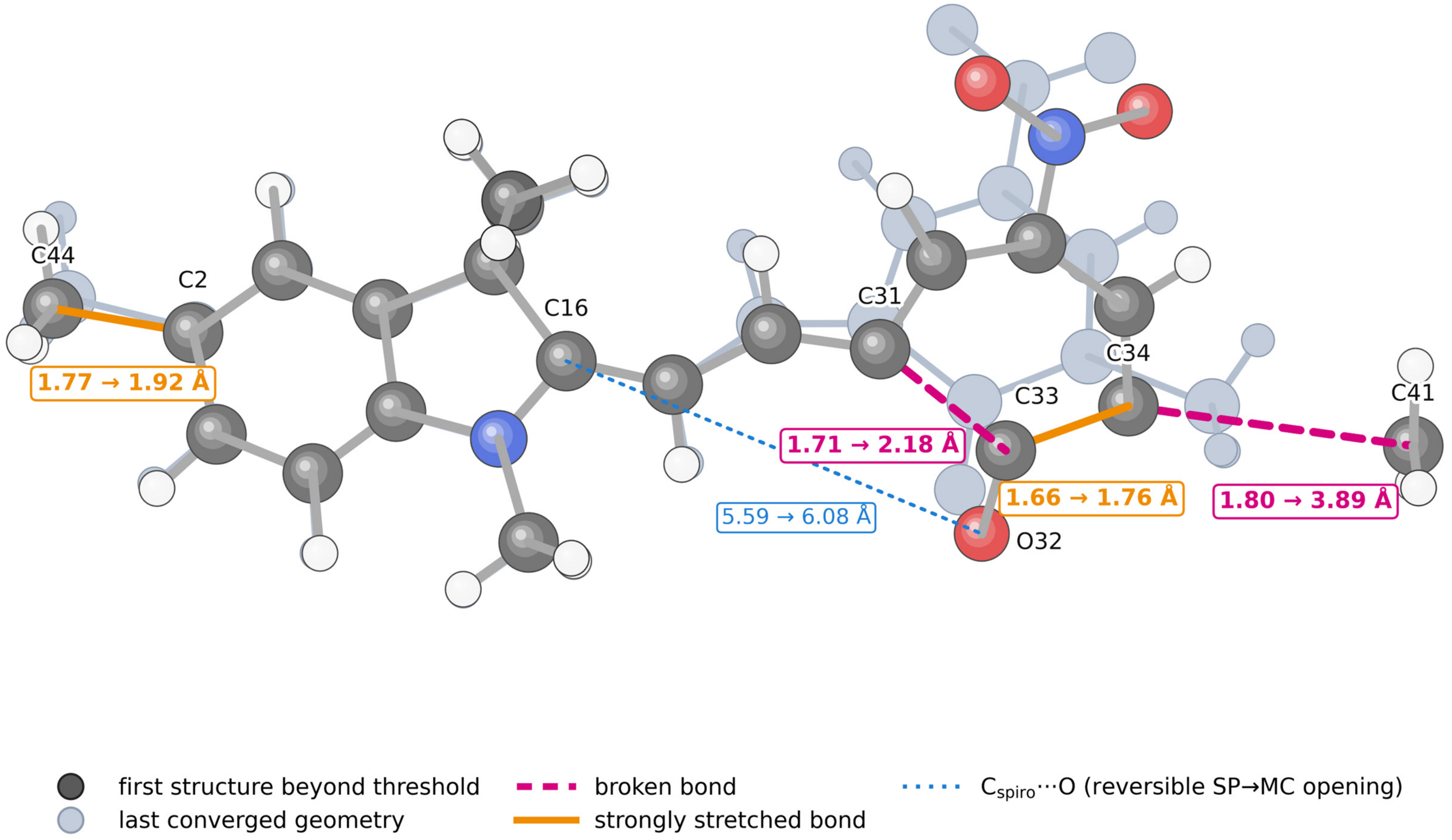

Figure S4. Overlay of the last converged geometry (pale blue) and the first structure beyond the sampled high-force damage boundary (dark grey) for SP–CH_3 at f = 0.070 a.u. (5.77 nN). Both geometries correspond to this applied force. Arrows give distances in these configurations, respectively, in Å. Magenta dashed lines mark damaged bonds; orange lines mark strongly stretched bonds. The blue dotted line marks the already-open spiro C–O pair. The dark configuration was reached during optimization and is not a converged stationary point. "Threshold" refers to the sampled damage boundary.

S9. Electronic energies and harmonic vibrational frequencies at zero force

The zero-force descriptor is defined as $\Delta E_r = E(MC_r, f = 0) - E(SP_s, f = 0)$. Each comparison uses the corresponding closed and relaxed structures of the same molecule in the same medium. Negative values place the mechanically reached relaxed MC below its closed SP reference at the reported level of theory. The energies describe the sampled conformers and do not establish global conformational equilibria or activation barriers.

Table S6. Absolute endpoint energies and relative electronic energies for the gas phase and thirteen COSMO media. Lowest harmonic vibrational frequencies are also listed.

Medium	$E(SP_s)$, E_h	$E(MC_r)$, E_h	ΔE_r, E_h	ΔE_r, kJ mol^{-1}	Relaxed MC	vmin (SP_s) cm^{-1}	vmin (MC_r) cm^{-1}
gas phase	−1220.682005234	−1220.690675792	−0.008670558	−22.76	TTC	+31.97	+23.53
n-heptane	−1220.695410298	−1220.702464552	−0.007054254	−18.52	TTC	+20.69	+20.69
CCl_4	−1220.697180712	−1220.705055495	−0.007874783	−20.68	TTC	+9.28	+19.87
toluene	−1220.697811635	−1220.703066795	−0.005255160	−13.80	CTC	+6.61	+18.14
o-xylene	−1220.699502937	−1220.697065376	+0.002437561	+6.40	CCC	+10.40	+25.70
diethyl ether	−1220.703049861	−1220.702134507	+0.000915354	+2.40	CCC	+7.93	+25.30
$CHCl_3$	−1220.704029709	−1220.703512566	+0.000517143	+1.36	CCC	+8.92	+25.22
MB	−1220.707088905	−1220.704939058	+0.002149847	+5.64	CCT	+10.36	+21.94
aniline	−1220.705846514	−1220.706267717	−0.000421203	−1.11	CCC	+8.72	+11.92
THF	−1220.706453513	−1220.704617111	+0.001836402	+4.82	CCT	+13.05	+21.80
1,2-DCA	−1220.707793296	−1220.706790417	+0.001002879	+2.63	CCT	+9.70	+21.60
pyridine	−1220.709841796	−1220.709370052	+0.000471744	+1.24	CCT	+10.37	+21.31
1-butanol	−1220.710804108	−1220.710945970	−0.000141862	−0.37	CCT	+12.91	+21.13
DMF	−1220.712192701	−1220.713212886	−0.001020185	−2.68	CCT	+10.77	+20.27

Energies are B3LYP/6-311G(d,p) electronic energies. COSMO entries use the PQS total energy including the dielectric contribution, without the separately printed OC correction. ΔE_r values in kJ mol^{-1} were calculated from unrounded energies using 1 E_h = 2625.49963948 kJ mol^{-1}. No zero-point or thermal correction is included. The conformer labels follow Table S1. vmin denotes the lowest harmonic vibrational frequency at zero external force, excluding translational and rotational modes. Harmonic vibrational analyses of the closed SP and zero-force-relaxed MC structures in the gas phase and all thirteen COSMO solvents yielded positive internal vibrational frequencies, confirming local-minimum character at the reported level of theory. A separate ZPE correction for the H-, NH_2- and Cl-substituted gas-phase structures is given in Table S8.

Table S7. Available gas-phase endpoint energies and lowest harmonic vibrational frequencies for the eleven substituted derivatives.

Z	$E(SP_s)$, E_h	$E(MC_r)$, E_h	ΔE_r, E_h	ΔE_r, kJ mol^{-1}	vmin (SP_s) cm^{-1}	vmin (MC_r) cm^{-1}
H	−1016.129092626	−1016.127843824	+0.001248802	+3.28	+24.70	+23.28
NH_2	−1071.499294389	−1071.496039301	+0.003255088	+8.55	+21.20	+21.84
Cl	−1475.749973043	−1475.750645319	−0.000672276	−1.77	+13.04	+23.56
NO_2	−1220.682005234	−1220.690675792	−0.008670558	−22.76	+31.97	+23.53
CHO	−1129.485731796	−1129.487088211	−0.001356415	−3.56	+13.00	+23.73
CN	−1108.394997979	−1108.397347444	−0.002349465	−6.17	+11.62	+23.97
Br	−3589.669574744	−3589.670242115	−0.000667371	−1.75	+10.15	+23.76
F	−1115.391772278	−1115.391885640	−0.000113362	−0.30	+16.94	+24.09
C≡CH	−1092.294199341	−1092.294879136	−0.000679795	−1.78	+19.13	+24.16
OH	−1091.369747904	−1091.369966706	−0.000218802	−0.57	+20.34	+23.23
$N(CH_3)_2$	−1150.127511438	−1150.127773151	−0.000261713	−0.69	+18.84	+21.98

ΔE_r compares the zero-force closed SP and relaxed MC structures. The H, NH_2 and Cl entries use the electronic energies and frequencies from the corresponding zero-force frequency outputs; their ZPE corrections are reported separately in Table S8. Table S7 contains electronic energies without ZPE. The complete substituent comparison covers all eleven derivatives: H, NH_2, Cl, NO_2, CHO, CN, Br, F, C≡CH, OH and $N(CH_3)_2$; all paired endpoint energies are listed above. The NO_2, CHO, CN, Br, F, C≡CH, OH and $N(CH_3)_2$ differences were calculated from the endpoint energies at the precision shown. vmin is the lowest harmonic vibrational frequency at f = 0, excluding translational and rotational modes; negative values denote imaginary frequencies. All listed vmin values are positive, confirming local-minimum character for the closed SP and zero-force-relaxed MC structures of all eleven gas-phase derivatives at the reported level of theory.

S9.1. Harmonic zero-point energy corrections for H, NH_2 and Cl

The effect of zero-point vibrational energy was evaluated for the gas-phase derivatives with X = Y = OH and Z = H, NH_2 or Cl. The closed SP and zero-force-relaxed MC structures were treated at B3LYP/6-311G(d,p). The full harmonic analyses contained 120 internal modes for each H and Cl structure and 126 for each NH_2 structure; all were positive. The ZPE values were taken from these analyses using unscaled harmonic frequencies.

$\Delta ZPE = ZPE(MC_r) - ZPE(SP_s)$; $\Delta E_{r,0} = \Delta E_r + \Delta ZPE$, at f = 0.

Table S8. Electronic energy differences and harmonic ZPE corrections for the gas-phase H-, NH_2- and Cl-substituted derivatives at zero external force.

Z	$ZPE(SP_s)$ kcal mol^{-1}	$ZPE(MC_r)$ kcal mol^{-1}	ΔE_r kJ mol^{-1}	ΔZPE kJ mol^{-1}	$\Delta E_{r,0}$ kJ mol^{-1}
H	213.547	213.003	+3.28	−2.28	+1.00
NH_2	223.894	223.404	+8.55	−2.05	+6.50
Cl	207.496	206.995	−1.77	−2.10	−3.86

Electronic energies and ZPE values are paired from the same frequency outputs; the corresponding electronic energies and lowest frequencies are listed in Table S7. Differences use 1 E_h = 2625.49963948 kJ mol^{-1} and 1 kcal mol^{-1} = 4.184 kJ mol^{-1}. Absolute ZPE values are reported at the output precision of 0.001 kcal mol^{-1}. Energy differences and sums were evaluated before rounding. $\Delta E_{r,0}$ includes harmonic ZPE only; finite-temperature and entropic contributions are excluded.

The ZPE corrections lower the relative MC energies by 2.05–2.28 kJ mol^{-1}. The signs and relative ordering of ΔE_r remain unchanged for these three sampled pairs: the relaxed MC lies above SP for H (+1.00 kJ mol^{-1}) and NH_2 (+6.50 kJ mol^{-1}), and below SP for Cl (−3.86 kJ mol^{-1}). This comparison assesses the zero-force energy differences of the sampled conformers. This ZPE comparison does not determine activation barriers or opening forces. Separate diffuse-basis and dispersion tests are reported below. Electronic stability was tested independently for the six fixed geometries listed in Section S9.4 and Table S11.

S9.2 Diffuse basis functions for NH_2 and Cl

Single-point B3LYP/6-311+G(d,p) energies were evaluated on the existing gas-phase B3LYP/6-311G(d,p) closed SP and zero-force-relaxed MC geometries for NH_2 and Cl. All entries are electronic energies; ZPE, thermal and dispersion corrections are excluded. The H derivative is not included in this diffuse-basis comparison.

Table S9. Diffuse-basis endpoint energies and changes in ΔE_r at zero external force.

Z	E(SP) 6-311+G(d,p) E_h	$E(MC_r)$ 6-311+G(d,p) E_h	ΔE_r 6-311G(d,p) kJ mol^{-1}	ΔE_r 6-311+G(d,p) kJ mol^{-1}	Change in ΔE_r kJ mol^{-1}
NH_2	-1071.519578427	-1071.516993107	+8.55	+6.79	−1.76
Cl	-1475.767733382	-1475.769298827	−1.77	−4.11	−2.35

Adding diffuse functions lowers ΔE_r by 1.76 kJ mol^{-1} for NH_2 and 2.35 kJ mol^{-1} for Cl, preserving the signs for these two pairs. The result is a basis-set sensitivity check on the sampled geometries; it does not supply reoptimized structures or revised opening forces.

S9.3 Focused dispersion energy comparison

Pairwise D3(BJ) energies were evaluated with simple-dftd3 1.6.0 for 443 existing B3LYP/6-311G(d,p) geometries: H 96, NH_2 203 and Cl 144. Standard B3LYP damping parameters were s_6 = 1, s_8 = 1.9889, a_1 = 0.3981 and a_2 = 4.4211; s_9 = 0 and the ATM term was omitted. The molecular geometries were retained. No ZPE or thermal correction was included.

Table S10. Changes in D3 energy and force-modified potential over the selected opening segments. All energy differences are in kJ mol^{-1}; later geometry minus earlier geometry.

Z	Optimizer iterates	Cspiro···O before → after Å	ΔE(D3)	ΔV without D3	ΔV with D3
H	10–22	1.7829 → 3.0657	+8.85	−407.30	−398.45
NH_2	12–28	1.7636 → 3.0685	+8.71	−329.71	−321.00
Cl	9–22	1.7532 → 3.1232	+10.80	−450.89	−440.09

The selected segment extends from the last iterate with a spiro C–O separation below 1.8 Å to the first at or above 3.0 Å. These markers describe the comparison and are not universal rupture thresholds. For every geometry, V = E(B3LYP) + E(D3) − fL was evaluated at nominal f = 0.065 a.u., with L in bohr. For NH_2 this is an assigned energy-evaluation parameter: the source input states 0.065 a.u., whereas the printed force vectors have magnitude 0.060 a.u. Thus the NH_2 result is conditional on the nominal-force assumption and does not independently verify a source optimization at 0.065 a.u.

The corrected potential decreases between every pair of successive sampled geometries in the selected segments. D3 therefore imposes an energetic penalty without reversing this sampled decrease. Optimizer iterates are nonstationary geometries and do not constitute a minimum-energy reaction path; these changes are not activation barriers. No dispersion-corrected opening force or substituent ranking is determined by this post hoc test.

S9.4 Electronic stability on selected fixed geometries

B3LYP/6-311G(d,p) electronic-stability tests were performed in Gaussian 16 Revision B.01 using Stable=Opt, SCF=(Tight,XQC), Int=UltraFine and NoSymm. The PQS geometries were retained. In all six completed tests the initial restricted solution was already stable under the perturbations considered, and no unrestricted conversion was required. These are electronic-stability tests, separate from harmonic-frequency analyses.

Table S11. Completed Gaussian electronic-stability checks on fixed PQS source-scan geometries.

Z	Source geometry	Source f a.u.	E(RB3LYP) E_h	Lowest printed stability eigenvalue	Restricted solution stable
H	Last closed	0.060	−1016.02386808	+0.0694306	Yes
H	First open	0.062	−1016.02540593	+0.0165911	Yes
NH_2	Last closed	0.064	−1071.38001150	+0.0676713	Yes
NH_2	First open	0.066	−1071.38117965	+0.0081683	Yes
Cl	Last closed	0.058	−1475.65273759	+0.0687770	Yes
Cl	First open	0.060	−1475.65578933	+0.0166570	Yes

The source geometries come from the additional 0.002 a.u. scans, distinct from the revised 0.001 a.u. profiles. The tests cover the last closed and first open geometries of each of H, NH_2 and Cl. Stability is established for these six sampled structures and is not inferred for every point of the source scans. Printed $\langle S^2 \rangle$ values associated with triplet stability eigenvectors are not assigned to the restricted ground state.

S10. Atom numbering for structural and electronic analyses

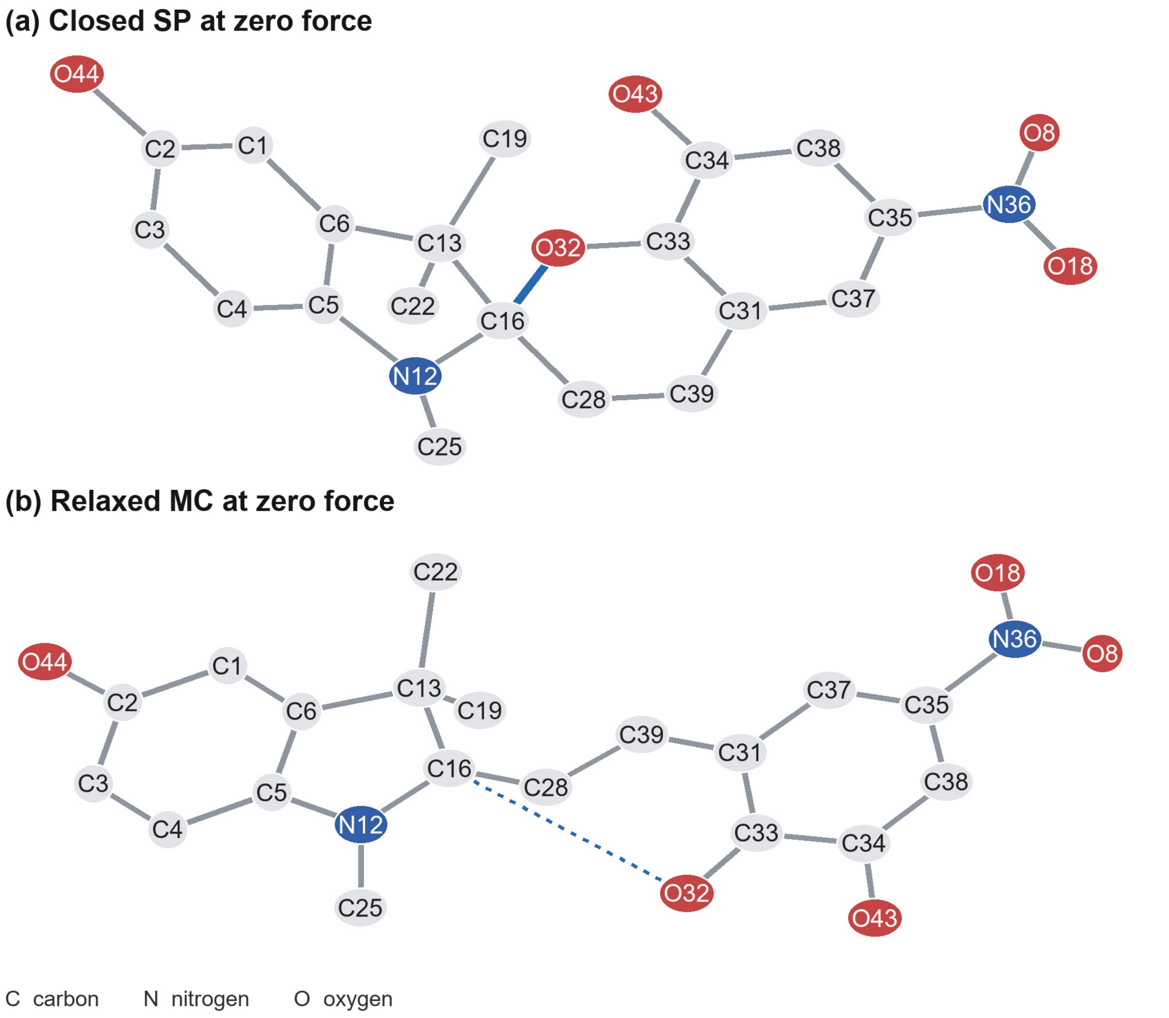

Figure S5. Atom numbering of (a) the closed SP–OH model (X = Y = OH, Z = NO_2) and (b) its zero-force-relaxed MC structure in the gas phase. Labels reproduce the PQS atom indices, which are unchanged between the two states. Hydrogen atoms are omitted for clarity. Sticks indicate connectivity without assigning bond order. Blue marks the C16–O32 bond in SP and, as a dotted line, the former C16···O32 pair in MC.

C16 and O32 identify the spiro carbon and oxygen used for the distances, Wiberg bond indices, oxygen CHELP charges, and σ/σ* NBO descriptors. N12 is the indoline nitrogen in LP(N12)→σ*(C16–O32). The labels also identify the atoms defining the torsions in Section S1 and the C5–C16–C25 reference plane used in Section S2.

The core labels for the spiro bond, indoline nitrogen, torsions, and nitrogen reference plane are retained in the H-, NH_2-, and Cl-substituted structures used for Table 1. Hydroxyl and substituent atom indices are model specific. Figure S4 concerns the distinct SP–CH_3 model and retains its own atom labels.

S11 Intramolecular hydroxy to spiro oxygen contact

Existing gas-phase closed-SP and zero-force-relaxed MC geometries were inspected for H, NH_2, Cl and NO_2. The adjacent hydroxy group points toward the spiro oxygen, which forms the phenolate part of MC after opening. The O–H···O contact exists in SP and becomes shorter and less bent after opening and relaxation. The NO_2 geometries correspond to the SP–OH model used in revised Figure 5.

Table S12. Geometry of the adjacent O–H···O contact in the four examined derivatives at zero external force.

Z	State	O–H<br>Å	H···O<br>Å	O···O<br>Å	∠O–H···O<br>°
H	SP	0.9672	2.1256	2.6765	114.58
H	MC	0.9776	1.9593	2.5976	120.65
NH_2	SP	0.9671	2.1430	2.6950	114.75
NH_2	MC	0.9789	1.9444	2.5876	120.93
Cl	SP	0.9674	2.1339	2.6806	114.25
Cl	MC	0.9784	1.9552	2.5944	120.66
NO_2	SP	0.9674	2.1249	2.6685	113.96
NO_2	MC	0.9770	1.9715	2.6023	120.07

The contact is O18–H41···O32 for H, NH_2 and Cl, and O43–H41···O32 for NO_2 in the respective output numbering; O32 is the spiro/phenolate oxygen. In the supplied SP–OH gas-phase scan the input specifies the force-bearing pair O43/O44, the oxygen atoms of the two hydroxy groups. O43 is bonded to C34 and H41, and O44 to C2 and H42.

Available NBO analyses show LP(O32)→σ*(O–H) donor–acceptor interactions. For NO_2, the printed E(2) contributions are 1.15 kcal mol^{-1} in SP and 5.13 kcal mol^{-1} in relaxed MC. For relaxed NH_2, the LP(1)(O32)→σ*(O–H) and LP(2)(O32)→σ*(O–H) contributions are 0.58 and 5.96 kcal mol^{-1}, respectively; for relaxed Cl, the corresponding LP(1) and LP(2) contributions are 0.55 and 5.64 kcal mol^{-1}. The printing threshold is 0.50 kcal mol^{-1}, so unprinted contributions are not assumed to vanish. E(2) is a perturbative donor–acceptor contribution, not an isolated hydrogen-bond energy. These contacts can contribute to MC stabilization, but the complete SP–OH/SP–CH_3 energy difference is not attributed to hydrogen bonding. Geometrical coverage here is limited to the four examined derivatives.

References

[12] Sheng, Y.; Leszczynski, J.; Garcia, A. A.; Rosario, R.; Gust, D.; Springer, J. Comprehensive theoretical study of the conversion reactions of spiropyrans: Substituent and solvent effects. J. Phys. Chem. B 2004, 108, 16233–16243. DOI: 10.1021/jp0488867.

[13] Cottone, G.; Noto, R.; La Manna, G. Theoretical study of spiropyran–merocyanine thermal isomerization. Chem. Phys. Lett. 2004, 388, 218–222. DOI: 10.1016/j.cplett.2004.03.016.

[18] Lin, Y.; Kouznetsova, T. B.; Foret, A. G.; Craig, S. L. Solvent polarity effects on the mechanochemistry of spiropyran ring opening. J. Am. Chem. Soc. 2024, 146, 3920–3925. DOI: 10.1021/jacs.3c11621.

[28] Rogers, R. A.; Rodier, A. R.; Stanley, J. A.; Douglas, N. A.; Li, X.; Brittain, W. J. A study of the spiropyran–merocyanine system using ion mobility-mass spectrometry: experimental support for the cisoid conformation. Chem. Commun. 2014, 50, 3424–3426. DOI: 10.1039/C3CC47697A.

[32] Kortekaas, L.; Browne, W. R. The evolution of spiropyran: fundamentals and progress of an extraordinarily versatile photochrome. Chem. Soc. Rev. 2019, 48, 3406–3424. DOI: 10.1039/C9CS00203K.

[34] Reed, A. E.; Curtiss, L. A.; Weinhold, F. Intermolecular interactions from a natural bond orbital, donor-acceptor viewpoint. Chem. Rev. 1988, 88, 899–926. DOI: 10.1021/cr00088a005.

[35] Yu, J.; Su, N. Q.; Yang, W. Describing Chemical Reactivity with Frontier Molecular Orbitalets. JACS Au 2022, 2, 1383–1394. DOI: 10.1021/jacsau.2c00085.

[36] Murugan, N. A.; Chakrabarti, S.; Ågren, H. Solvent dependence of structure, charge distribution, and absorption spectrum in the photochromic merocyanine–spiropyran pair. J. Phys. Chem. B 2011, 115, 4025–4032. DOI: 10.1021/jp2004612.

[37] Balasubramanian, G.; Schulte, J.; Müller-Plathe, F.; Böhm, M. C. Structural and thermochemical properties of a photoresponsive spiropyran and merocyanine pair: Basis set and solvent dependence in density functional predictions. Chem. Phys. Lett. 2012, 554, 60–66. DOI: 10.1016/j.cplett.2012.10.014.

[40] Eilmes, A. Spiropyran to merocyanine conversion: explicit versus implicit solvent modeling. J. Phys. Chem. A 2013, 117, 2629–2635. DOI: 10.1021/jp3117209.

[41] Behera, S.; Haldar, R.; Senapati, S.; Khan, A.; Majumdar, B. B.; De, S.; Maity, S. Interplay of solvent polarity and hydrogen bonding in thermal isomerization of spiropyran. J. Photochem. Photobiol. A: Chem. 2026, 472, 116727. DOI: 10.1016/j.jphotochem.2025.116727.

[46] Klamt, A.; Schüürmann, G. COSMO: A new approach to dielectric screening in solvents with explicit expressions for the screening energy and its gradient. J. Chem. Soc., Perkin Trans. 2 1993, 799–805. DOI: 10.1039/P29930000799.
